# Combinatorial biosynthesis of novel gentamicin derivatives with nonsense mutation readthrough activity and low cytotoxicity

**DOI:** 10.3389/fphar.2025.1575840

**Published:** 2025-04-24

**Authors:** Lihua Yang, Hang Zhai, Tingting Tian, Botong Liu, Xianpu Ni, Huanzhang Xia

**Affiliations:** School of Life Science and Biopharmaceutics, Shenyang Pharmaceutical University, Shenyang, China

**Keywords:** combinatorial biosynthesis, aminoglycoside, glycosyltransferase, premature termination codon readthrough, chemical compound

## Abstract

**Background:**

Aminoglycosides (AGs) are one of the initial classes of antibiotics that have been used clinically and possess broad spectrum of activity. Nevertheless, their clinical utilization is restricted by safety issues associated with nephrotoxicity and ototoxicity.

**Methods:**

Glycosyltransferase (GT) KanM2 was introduced into *M. echinospora* to produce the gentamicin derivatives, in which a kanosamine sugar ring was introduced to replace the garosamine. The premature termination codon (PTC) readthrough activity of genkamicins (GKs) was compared using dual luciferase reporter assay. The toxicity of GK was assessed *in vitro* in HEK-293 and NCI-H1299 cells and determined based on cell viability calculated after 48 h of treatment with different concentrations of the compounds. The NCI-H1299 cells harbouring the R213X nonsense mutation were treated with different concentrations of the derivatives to compare their expression of p53 proteins. The expression of p53 and its downstream targets p21 and BAX was detected using Western blotting and qRT-PCR in NCI-H1299 cells containing the R213X nonsense mutation treated with different concentrations of GK-Ae and G418. Finally, immunofluorescence and flow cytometry were used to determine the subcellular localization of full-length p53 protein induced by GK-Ae treatment and its effect on apoptosis in cancer cells.

**Results:**

Eight gentamicin derivatives were obtained in this study. GK-Ae displayed similar PTC readthrough activity and reduced toxicity compared to natural aminoglycoside G418. Moreover, GK-Ae increased the levels of both p53 and its downstream targets p21 and BAX, and promoted apoptosis of cancer cells.

**Conclusion:**

These results demonstrate the potential of combinatorial biosynthesis to increase the diversity of structures of AGs and provide directions for the development of new AGs with low toxicity and high PTC readthrough activity.

## 1 Introduction

It is reported that the cause of over 2,400 different genetic diseases in involved in at least one nonsense mutation (Mort et al., 2008; Linde and Kerem, 2008), such as cystic fibrosis (Peltz et al., 2013), Duchenne muscular dystrophy (Nguengang et al., 2020), Spinal muscular atrophy (Li et al., 2019), and tumour suppressor PTEN and p53 mutations caused cancers (Ferguson et al., 2019; Luna et al., 2021). Nonsense mutations introduce a premature termination codon (PTC) into the mRNA, which prematurely terminates translation to produce a truncated protein when the ribosome reaches the PTC site. This truncated protein might lose its original function or have a dominant negative effect (Guissart et al., 2020; Sylvain et al., 2002). As the most known gene associated with cancer discovered in the year 1979, p53, a tumour suppressor, has been found to fulfil crucial functions in controlling cell proliferation, senescence, DNA repair and cell death through both non-transcriptional and transcriptional activities to modulate specific gene expression in response to stimuli (Mao and Jiang, 2023; Lane and Levine, 2010; Lim et al., 2023). Transcriptional activation of target genes P21 and Bax, which are involved in cell cycle control and apoptosis, is critical for the role of p53 in tumor suppression (Deisenroth and Zhang, 2010; Bursac et al., 2014). The p53 protein synthesized in the cytoplasm is transported to the nucleus, and the nuclear transportation is mediated by nuclear localization signal and the tetramerization domain in the C-terminus of p53 (Liang and Clarke, 2001). Mutation of p53 is the most prevalent genetic alternation in cancer, and approximately 50% of human cancers display the high mutation of p53 (Kennedy and Lowe, 2022; Zhou et al., 2019). Nonsense mutations account for approximately 10% fraction of the p53 mutations, once p53 is inactivated due to a mutation or deletion of the gene, normal cells might lose control of their growth, eventually leading to cancer (Strandgren and Wiman, 2024).

The most widespread strategy for treating nonsense mutations is drug-induced ribosomal readthrough of PTCs to restore the production of the full-length protein. These molecules promote the incorporation of a nearcognate tRNA at the PTC position to continuously translating and synthesizing full-length proteins in the correct reading frame. The PTC readthrough therapy is not gene-specific and can thus be applied to treat various genetic diseases.

**FIGURE 1:**
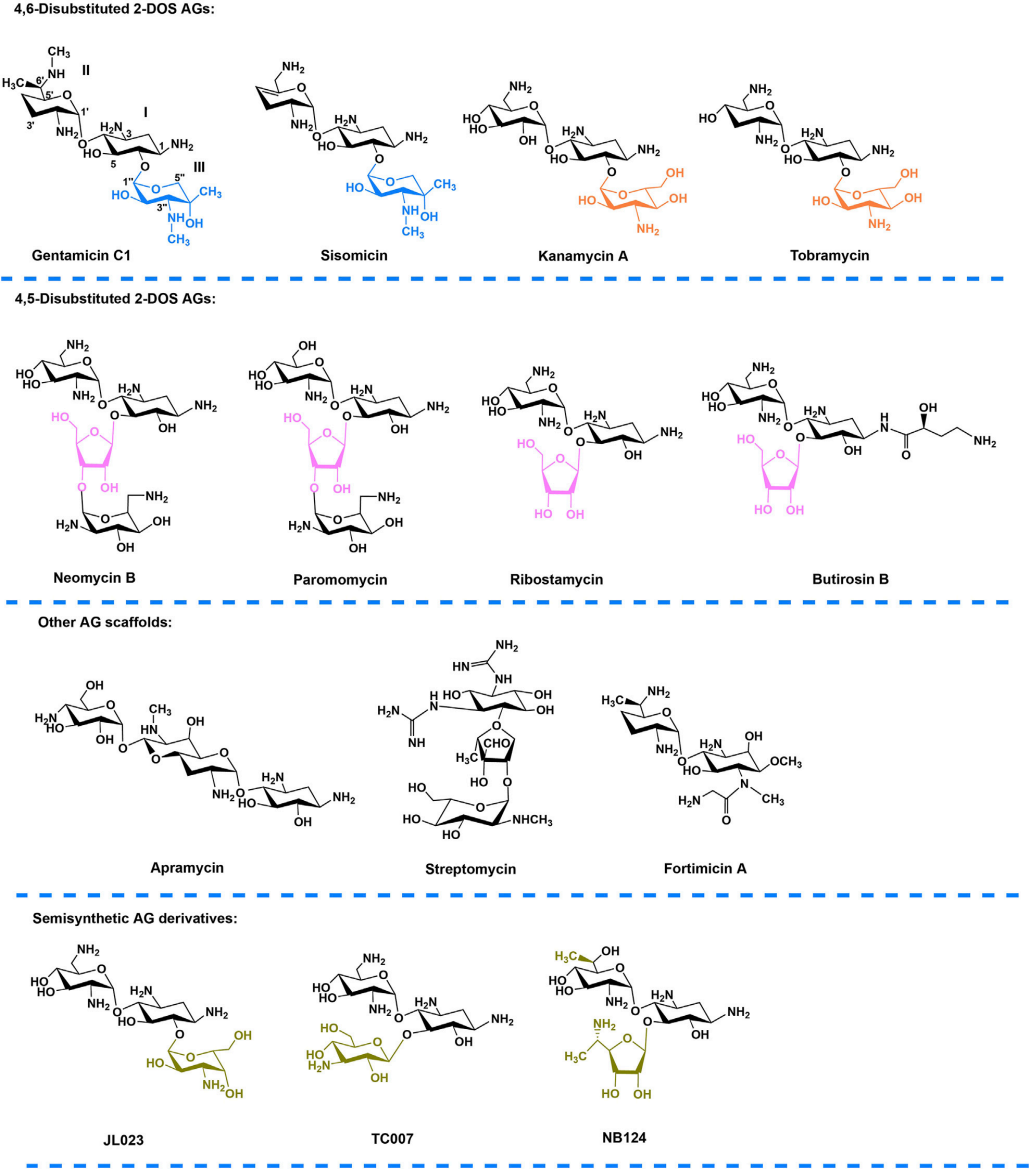
Chemical structures of natural AGs and their semisynthetic derivatives. Blue, orange, and purple moieties represent the ring III (xylose, glucose, and ribose respectively) in different family AGs. Gold indicates the chemically modified features of the semisynthetic AGs. Annotation to carbon and ring numbers is shown in gentamicin C1.

Aminoglycosides (AGs) is a class of broad-spectrum antibiotic agents used extensively in clinics against Gram-positive and Gram-negative bacterial pathogens (Just et al., 1985; Shields et al., 2016; Zhang et al., 2017). AGs has shown the potential in the treatment of human genetic diseases (Zingman et al., 2007; Floquet et al., 2011). In 1985, G418 and paromomycin were firstly discovered to induce PTC readthrough in the COS-7 cells (Burke and Mogg, 1985). After this, gentamicin, neomycin, kanamycin, amikacin, arbekacin, and lividomycin, which all belong to the AG family, have been demonstrated to show different PTC readthrough efficacies (Howard et al., 1996; Howard et al., 2004; Kuschal et al., 2015; Lee and Dougherty, 2012). Although G418 is considered as one of the strongest readthrough inducers, it is also the most toxic AG which cannot be used for therapy. Gentamicin is the principal molecule currently being tested in clinical trials, but the potential ototoxicity and nephrotoxicity restrict its long-term use (Selimoglu, 2007; Wargo and Edwards, 2014). Targeted modification or structural design of AGs with reduced toxicity and increased readthrough activity are therefore desirable to further improve long-term efficacy and safety. To date, three classes of AG derivatives have been developed (Figure 1), including “JL derivatives” (derived from kanamycin) (Mattis et al., 2006), “TC” derivatives (derived from neomycin) (Mattis et al., 2009) and “NB” derivatives (derived from paromomycin or G418) (Nudelman et al., 2006). The research on AG derivatives to improve potency and reduce toxicity is promising.

**FIGURE 2:**
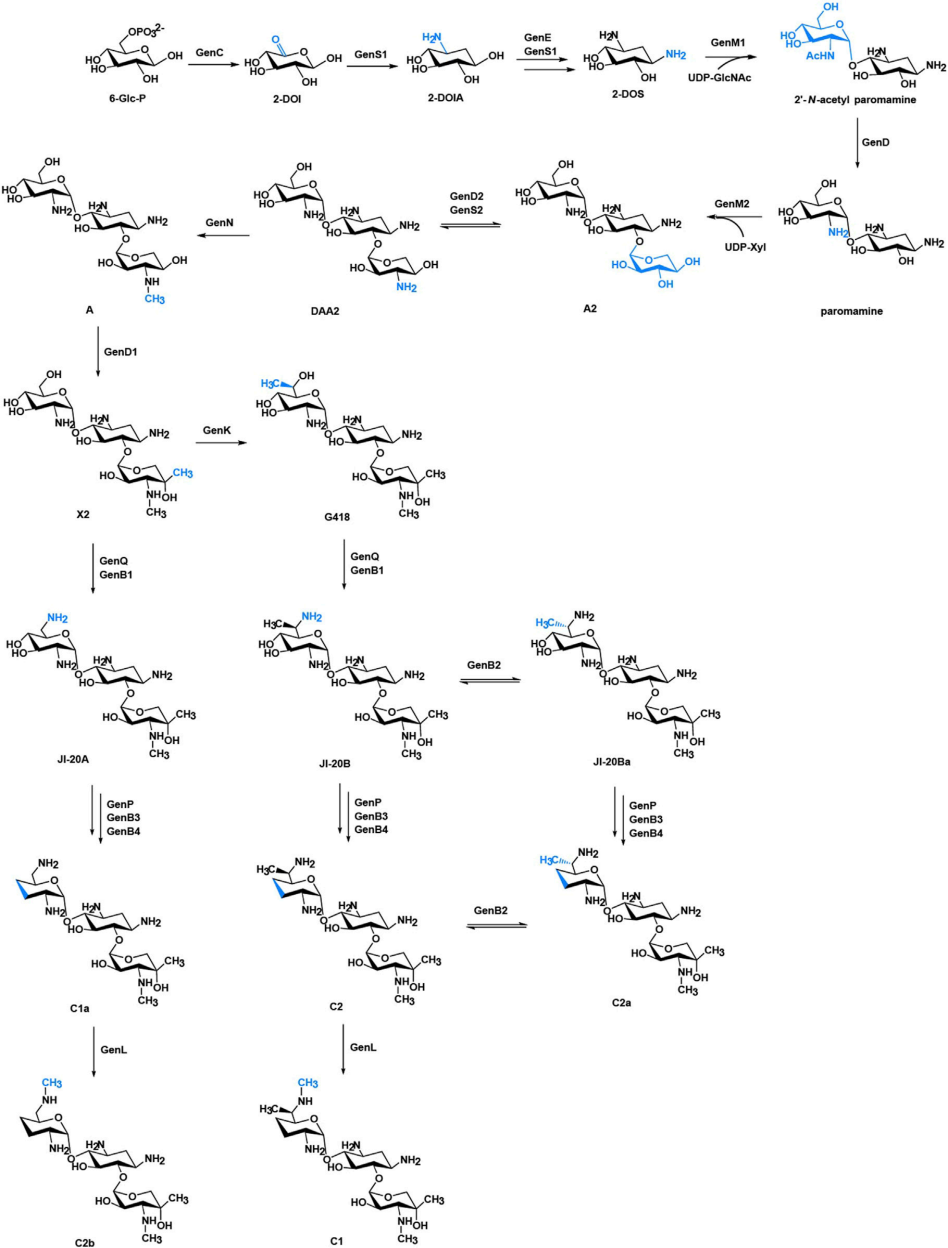
Biosynthetic pathway of gentamicins. The structural changes are marked in blue. Abbreviations: 6-Glc-P, glucose 6-phosphate; 2-DOI, 2-deoxy-scyllo-inosose; 2-DOIA, 2-deoxy-scyllo-inosamine; 2-DOS, 2-deoxystreptamine; UDP-GlcNAc, uridine 5′-diphospho-D-2-N-acetylglucosamine; UDP-Xyl, UDP-xylose.

According to the substitution site of aminocyclitol, AGs can be categorized into three subtypes: 1) 4,6-disubstituted 2-deoxystreptamine (2-DOS), such as kanamycin, gentamycin, and tobramycin; 2) 4,5-disubstituted 2-DOS, such as ribostamycin, neomycin, and paromomycin; and 3) other atypical structural aminoglycosides, such as streptomycin with a streptidine ring as the central aminocyclitol and fortimicin with a 1,4-diaminocyclitol, apramycin with 2-DOS monosubstituted in position 4 (Figure 1) (Zingman et al., 2007; Ennifar et al., 2013).Of note, gentamicin (Figure 1) containing the core 2-DOS moiety with the amino-sugars purpurosamine and garosamine attached at positions C4 and C6 is a highly modified aminoglycoside antibiotic. The different methylation leads to the different biological activities of the components. The biosynthetic pathway of gentamicin has been studied through *in-vivo* knockout and *in-vitro* heterologous expression since the biosynthetic gene cluster of gentamicin was identified more than a decade ago (Figure 2) (Park et al., 2008; Guo et al., 2014). Methylation is defined by the selectivity of the three methyltransferases, namely, GenK, GenN, and GenD1, and there is a network of methylation comprised in gentamicin pathway (Palacios-Muñoz and Ewer, 2018). Methyltransferase GenK was proved as a key enzyme at the biosynthetic branch point in a previous work (Kim et al., 2013). Kanamycin (Figure 1) is another representative 4,6-disubstituted 2-DOS AGs along with the gentamicin. One of the structural features of kanamycin is the kanosamine (3-amino-3-deoxy-D-glucose), which is connected to C6 of the 2-DOS moiety. The synthetic routes of kanamycin are very similar to gentamicin, and the homology among their functional genes is relatively high (Supplementary Figure S1) (Park et al., 2011).

The diversity of sugar moieties led to different bioactivity of AGs, and the glycosylation has been widely applied in developing new drugs (Kren and Rezanka, 2008). The substrate flexibility of key enzymes has contributed to the complex network both in gentamicin and kanamycin biosynthetic pathway, which can be used for combinatorial biosynthesis. Based on this, we introduced KanM2 into gentamicin biosynthetic pathway to produce a series of gentamicin derivatives, in which a garosamine was replaced with kanosamine as the ring III. During the preparation of this work, Jian et al. reported an approach exploiting the bacterial biosynthetic mechanism of gentamicins by the glycodiversification of gentamicins via swapping the glycosyltransferase (GT) in their producer with the GT from kanamycins biosynthesis pathway, leading to the production of some novel AG, known as genkamicins (GKs) (Jian et al., 2024). In this study, we marked these new structures by the same way. The R213X mutation (a UGA premature termination codon replaces CGA) is the most frequent p53 nonsense mutation in human tumours. Therefore, it is necessary to explore whether the AGs influences the treatment of cancer with R213X mutation, to explore whether full-length p53 proteins induced by GK-Ae produce normal function, and to explore safety issues related to nephrotoxicity and ototoxicity. The present study was carried out based on these issues in the expectation of obtaining AG derivatives with lower toxicity for the treatment of cancer.

## 2 Materials and methods

### 2.1 Construction of phylogenetic tree

To understand the relationship of AGs, a phylogenetic tree was constructed using ape package in R (Paradis et al., 2019) to analyse the 28 proteins known in charge of glycosylation involved in AGs biosynthesis (Supplementary Table S1). The tree was constructed with the neighbor-joining method having the number of bootstrap replications set to 1000.

### 2.2 Bacterial strains and culture conditions

*E. coli* strains were grown in Luria–Bertani liquid medium or solid medium (1.0% yeast extract (LP0021, Oxoid, Basingstoke, UK), 0.5% tryptone (LP0042, Oxoid, UK), 1.0% sodium chloride (NaCl, 1001-0624, Yongda Chemical, Tianjin, China)) at 37°C with the appropriate antibiotics including at a final concentration of ampicillin (final concentration: 100 µg/mL, A408319, Aladdin, Shanghai, China), apramycin (final concentration: 50 µg/mL, A425315, Aladdin, China), chloramphenicol (final concentration: 25 µg/mL, C408156, Aladdin, China), or kanamycin (25 µg/mL, K331597, Aladdin, China). Supplementary Table S2 listed all the bacterial strains used in this work.

The wild-type and mutant strains of *M. echinospora* ATCC 15835 were maintained in the growth medium at 34°C for sporulation (Chen et al., 2020). For the metabolite production, the wild-type strain of *M. echinospora* ATCC15835 and its mutants were maintained in seed culture at 34°C after shaking at 220 rpm for 36 h, then transferred 10% (v/v) into the fermentation culture and incubated for 5 days (Chen et al., 2020).

### 2.3 Construction of mutant strains

For gene disruption, flanking regions of *gen*M2 were amplified from the genomic DNA of *M. echinospora* ATCC15835 using Pfu DNA polymerase (B500014, Sangon Biotech, Shanghai, China) and cloned into the pPT2925 plasmid (which was derived from pIJ2925), then cloned into the *E. coli*-*M. echinospora* vector pKC1139 between *Eco*RI and *Hin*dIII sites to obtain the gene disruption plasmid pKCD2D1. The obtained plasmid pKCD2D1 was thereafter introduced into the wild-type and Δ*gen*K strains by conjugation and mutants screening following a previous study (Chen et al., 2020) for the generation of Δ*gen*M2 and Δ*gen*KΔ*gen*M2 strains. Supplementary Tables S2, S3 list the plasmids and primers used in this study, respectively.

The promoter *kas*Op* and gene *kan*M2 were amplified from the pJY813 and *S. kanamyceticus* CGMCC4.1441 using Pfu DNA polymerase respectively, and then ligated together by overlapping PCR. The *kas*Op*-*kan*M2 fragment were subsequently cloned into pKCD2D1 plasmid between *Eco*RV and *Xba*I sites to obtain the gene expression plasmid pKCM2L.

Two flanking regions of *gen*M2-*gen*D1 were additionally amplified from the genomic DNA of *M. echinospora* ATCC15835 using Pfu DNA polymerase and cloned into the pPT2925 plasmid. Then, the two DNA fragments and *kas*Op*-*kan*M2 were cloned into the *E. coli*–*M. echinospora* vector pKC1139 between *Eco*RI and *Hin*dIII sites to obtain the gene expression plasmid pKCM2LS2.

In the same way, pKCM2L and pKCM2LS2 plasmids were introduced into the wild-type strain and Δ*gen*K to generate the strains of Δ*gen*M2::*kan*M2, Δ*gen*KΔ*gen*M2::*kan*M2, Δ*gen*M2Δ*gen*D1::*kan*M2 and Δ*gen*KΔ*gen*M2Δ*gen*D1::*kan*M2. All mutants were confirmed by PCR amplification using the primers (Supplementary Table S3).

### 2.4 Extraction, isolation, purification, and analysis of metabolites

#### 2.4.1 Extraction, isolation, and purification of metabolites

Extraction, isolation and purification of metabolites were performed to obtain more pure metabolites. The fermentation broth was maintained at pH 2.0 with sulfuric acid (H_2_SO_4_, 101502-0003, Yongda Chemical, China), stirred for 3 h and centrifuged (10,000 × g, 10 min) at 4°C. Each supernatant was subsequently readjusted to pH 7.0 with sodium hydroxide (NaOH, 1001-0746, Yongda Chemical, China) and centrifuged again (10,000 × g, 10 min). Strong acidic resin 732 (Amicogen Co., Ltd., Jining, China) was used to absorb the supernatant and then eluted with 2.0 mol/L ammonia solution (NH_4_OH, 1001-0095, Yongda Chemical, China) with passing through strong basic 711 resin (Amicogen Co., Ltd., China) for discoloration.

The crude extracts were freeze-dried, redissolved in Milli-Q water and filtered through 0.22 μm microporous membrane before subjection to high-performance liquid chromatography (HPLC) analysis and purification of compounds. Thereafter, the components were separated on the ion-exchange resin D152 column (Amicogen Co., Ltd., China), and the products were eluted by a gradient of NH_4_OH (from 0.01 to 1.0 mol/L). Fractions containing single component were combined and concentrated by reduced pressure distillation, lyophilized, and analysed by mass spectrometry (MS) and nuclear magnetic resonance (NMR).

#### 2.4.2 Metabolites analysis and mono component purification with HPLC-evaporative light scattering detection (ELSD)

Metabolites analysis and mono component purification from crude extract were conducted using an evaporative light scattering detector (MODEL 100, SofTA, United States) connected with a liquid chromatography (LC-6AD, Shimadzu, Kyodo, Japan) fitted with an Ultimate LP-C18 column (4.6 × 250 mm; Welch Materials, Inc., West Haven, Connecticut, United States). The mobile phase was 0.2 mol/L trifluoroacetic acid (T818781, Macklin, Shanghai, China)-methanol (1007-0048, Yongda Chemical, China) (92:8, v/v) used at a flow rate of 0.8 mL/min. Targeted fractions were collected and freeze-dried.

#### 2.4.3 MS and NMR analysis

MS analysis was performed on Waters G2-S Q-tof mass spectrometer (Waters Corporation, Milford, Massachusetts, United States). The MS analysis was performed in positive mode. In detail, the compounds were purified by freeze-drying to 5–10 mg and were dissolved in 500 μL of deuterium oxide (D_2_O). The 1D (^1^H and ^13^C) and 2D (^1^H–^1^H COSY, HSQC, HMBC and NOESY) NMR analyses were performed on Bruker AV600 NMR spectrometer (Bruker, Billerica, Massachusetts, United States) at 600 MHz (^1^H) and 151 MHz (^13^C). Chemical shifts were reported in ppm, and NMR data processing was performed by using MestReNova software (version 15.1, Mestrelab, Santiago de Compostela, Spain).

### 2.5 Cell culture, transfection and drug treatment

Human embryonic kidney 293 (HEK 293) cells (CL-0001, Procell, Wuhan, China) were grown in DMEM (11996025, Gibco, Waltham, Massachusetts, United States), and the lung adenocarcinoma cell line NCI-H1299 (p53-null) was derived from the original cell line (CL-0165, Procell, China) based on an existing protocol (Mitsudomi et al., 1992) and were grown in Roswell Park Memorial Institute-1640 medium (11875168, Gibco, United States). Both media were added with 10% FBS (C0234, Beyotime, China) and cells were maintained in a 37°C culture incubator with the presence of 5% CO_2_.

For the transfection, the cells were inoculated into six-well plates at 3 × 10^6^ cells/well. 24 h later, the mixture of the reporter plasmid and the transfection reagent (Lipo8000™, C0533, Beyotime, China) were transiently transfected into the cells following the instruction. Next day, cells containing different reporter plasmids were processed with the indicated compounds for 48 h and harvested for following analysis.

In the dual-luciferase reporter assay for readthrough, the plasmid pSiCheck2 with the *Renilla* and firefly luciferase gene was used as backbone and empty vector. Thereafter, the DNA fragments including the tested nonsense mutation 213X (TGA) or the corresponding wild-type codon (CGA), along with four upstream and three downstream flanking codons of human p53 coding sequence, were inserted into the open reading frame of *Renilla* luciferase gene after the initiation codon to generate pSiCheck2-213X and pSiCheck2-WT constructs.

Additionally, the plasmid pCDH-CMV-MCS-EF1-copGFP with the CMV promoter served as backbone and empty vector. The human p53 coding sequence with a nonsense mutation at codon 213 and the wild-type p53 coding sequence were inserted downstream of the CMV promoter for the generation of the constructs pCMV-213X and pCMV-WT, respectively. The two constructs were transiently transfected into NCI-H1299 cells for the following assays. All constructs were confirmed by sequencing.

### 2.6 Dual-luciferase reporter assay

This reporter assay system contained a Firefly luciferase gene, which positioned upstream of the PTC and expressed constitutively, and a Renilla luciferase gene, with the CDS interrupted by R213X mutation and expressed only when PTC readthrough was induced.

HEK293 cells were transiently transfected with dual-luciferase reporter constructs (pSiCheck2-213X and pSiCheck2-WT) and then incubated in white 96-well plates with various concentrations of test drugs. After 48 h, cells were lysed using lysis buffer and centrifuged at 10,000 g for 5 min to collect the clear supernatants. Dual-luciferase activity was assayed with the Dual Luciferase Assay System (RG058M, Beyotime, China) by the FLX800 multiscan spectrum microplate reader (BioTek, Winooski, Vermont, United States) following the protocol. The readthrough activity was defined as the ratio of Renilla to Firefly relative luciferase units obtained from the PTC-containing construct normalized to the ratio of Renilla to Firefly obtained from the corresponding WT construct.

### 2.7 Cytotoxicity assay

A CCK-8 assay kit (C0037, Beyotime, China) was used to assess the *in-vitro* cytotoxicity of GKs. Concretely, both HEK-293 and NCI-H1299 cells grown in 96-well plates overnight were treated with various concentrations of the tested aminoglycosides for 48 h. Then, the cells were incubated after adding CCK-8 working solution (10 µL) for 2 h. The optical density (OD) value at 450 nm was determined with the microplate reader (Multiskan MK3, Thermo Scientific, Waltham, Massachusetts, United States). Cell viability was calculated as per the formula:

$$\text{Cell viability }\left(\%\right)=\frac{\left({\text{OD}}_{\text{treated}}-{\text{OD}}_{\text{empty}}\right)}{\left({\text{OD}}_{\text{untreated}}-{\text{OD}}_{\text{empty}}\right)}\times 100\%$$

### 2.8 Western blotting

Western blotting was used to test the production of the full-length p53 protein. Following the transient transfection of the constructs (pCMV-213X and pCMV-WT), different concentrations of the drugs were added to incubate the NCI-H1299 cells in the six-well plates for 48 h. Thereafter, NCI-H1299 cells were harvested and lysed with ice-cold RIPA buffer (G2002, Servicebio, Wuhan, China) supplemented with 1% protease inhibitor (G2006, Servicebio, China) for 10 min. The cell lysates were subsequently centrifuged at 10,000 g for 10 min at 4°C, and clear supernatants were collected. Total proteins were quantified with Bradford reagent (5000201, Bio-Rad, Hercules, California, United States) and the extracts were denatured by incubating in SDS loading buffer for 10 min at 100°C.

Then, 30 µg of total proteins were isolated by 12% SDS polyacrylamide gel electrophoresis (SDS-PAGE, P0012A, Beyotime, China) and moved onto the PVDF membranes (1620177, Bio-Rad, United States) following the manual. Membranes were blocked with 5% skimmed milk in PBS containing 0.1% Tween 20 (PBST) at room temperature for 2 h and then incubated overnight at 4°C with the primary antibodies against p53 (60283-2-Ig, Proteintech, Rosemont, Illinois, United States) and the loading control β-actin (GB11001, Servicebio, China). Then, the horseradish peroxide (HRP)-conjugated secondary antibodies goat-anti-mouse IgG (A0216, Beyotime, China) or goat-anti-rabbit IgG (ZB-2301, ZSGB-BIO, Beijing, China) were applied to incubate with the membranes for 1 h at room temperature to detect primary antibodies. The membranes were washed three times with PBST in each step.

For visualization, the membranes were treated with the ECL detection kit (G2020-1, Servicebio, China) and exposed in ChemiDOC XRS + imaging system (Bio-Rad, United States). Finally, the densitometry of the protein bands was quantified with ImageJ software (version 5.0, Bio-Rad, United States).

### 2.9 RNA extraction and qRT-PCR

To assess whether AGs affects the RNA stability of p53 and its transcriptional target genes p21 and Bax, NCI-H1299 (R213X) cells were pre-treated with different concentrations of compounds for 48 h. The total RNA was then extracted by using the RNA extraction kit (DP424, TIANGEN, Beijing, China) and reverse transcribed into cDNA with HiScript II Q RT SuperMix (R222, Vazyme, Nanjing, China) following the protocol. The qRT-PCR was performed on the MA-688 Real-Time Quantitative Thermal Cycler (Molarray, Suzhou, China) using Blastaq™ 2×qPCR Mastermix (ABS-G891, Amyjet Scientific, Wuhan, China). The primers for the amplification of the specific cDNA sequences were shown in Supplementary Table S3. With β-actin as endogenous control, relative mRNA levels were calculated by the 2^−ΔΔCT^ method from three independent sets (Livak and Schmittgen, 2001).

### 2.10 Immunofluorescence and confocal imaging

NCI-H1299 (pCMV-213X) cells were inoculated on the glass slides of a 24-well plate and incubated at 37°C overnight, and treated with different concentrations of AGs for 48 h. Subsequently, cells were fixed by PBS containing 4% paraformaldehyde (P0099, Beyotime, China), permeabilized with 0.5% Triton X-100 (ST1722, Beyotime, China) for 10 min, and blocked with 5% bovine serum albumin (ST023, Beyotime, China) for 1 h at 25°C. Subsequently, the cells were incubated with the antibody against p53 (ENZ-ABS653-0200, Enzo Biochem Inc., New York, United States) at 4°C overnight, rinsed three times with PBS and incubated with Cy3-conjugated anti-mouse IgG (SA00009-1, Proteintech, United States) at 25°C for 1 h. Finally, the cells were mounted using anti-fade mounting medium with 4′,6-diamidino-2-phenylindole (DAPI, S2110, Solarbio, China) and analysed using a confocal laser scanning microscope (Stellaris 5, Leica, Wetzlar, Germany). Image analysis and cell counting were performed using at least three coverslips for each experimental group with the help of ImageJ software.

### 2.11 Flow cytometry

A general marker of apoptotic cells is phosphatidylserine (PS) exposure on the cell surface. We assessed apoptosis by flow cytometry using fluorochrome-labelled Annexin V, a phospholipid-binding protein with a high affinity for PS.

NCI-H1299 cells transfected with the indicated vectors (pCMV-213X and pCMV-WT) or the empty vector were seeded at a concentration of 3 × 10^5^ cells/well in six-well plates in a volume of 2 mL. Next day, cells were treated with different concentrations of test compounds for the 48-h incubation. Thereafter, the attached and floating cells were harvested using non-EDTA trypsin (C0205, Beyotime, China) and washed with PBS. Cells were then resuspended in 500 μL binding buffer and incubated together with 5 μL Annexin V-APC and 5 μL propidium iodide provided by the assay kit (A214-01, Vazyme, China) for 10 min at room temperature away from light. Cytoflex flow cytometer (Beckman Coulter, Indianapolis, Indiana, United States) was used to quantify the apoptotic cells.

### 2.12 Statistical analysis

Data of three independent assays were expressed as mean ± standard error of the mean (SEM). All statistical analyses were performed using GraphPad Prism (version 10, GraphPad Software, Inc., La Jolla, California, United States). For assays with multiple conditions, one-way or two-way ANOVA followed by Tukey’s or Dunnett’s testing for multiple comparisons were applied. The data with a *p* < 0.05 denoted a statistical significance (*p < 0.05, **p < 0.01, ***p < 0.001, ****p < 0.0001).

## 3 Results

### 3.1 The construction of phylogenetic tree

**FIGURE 3:**
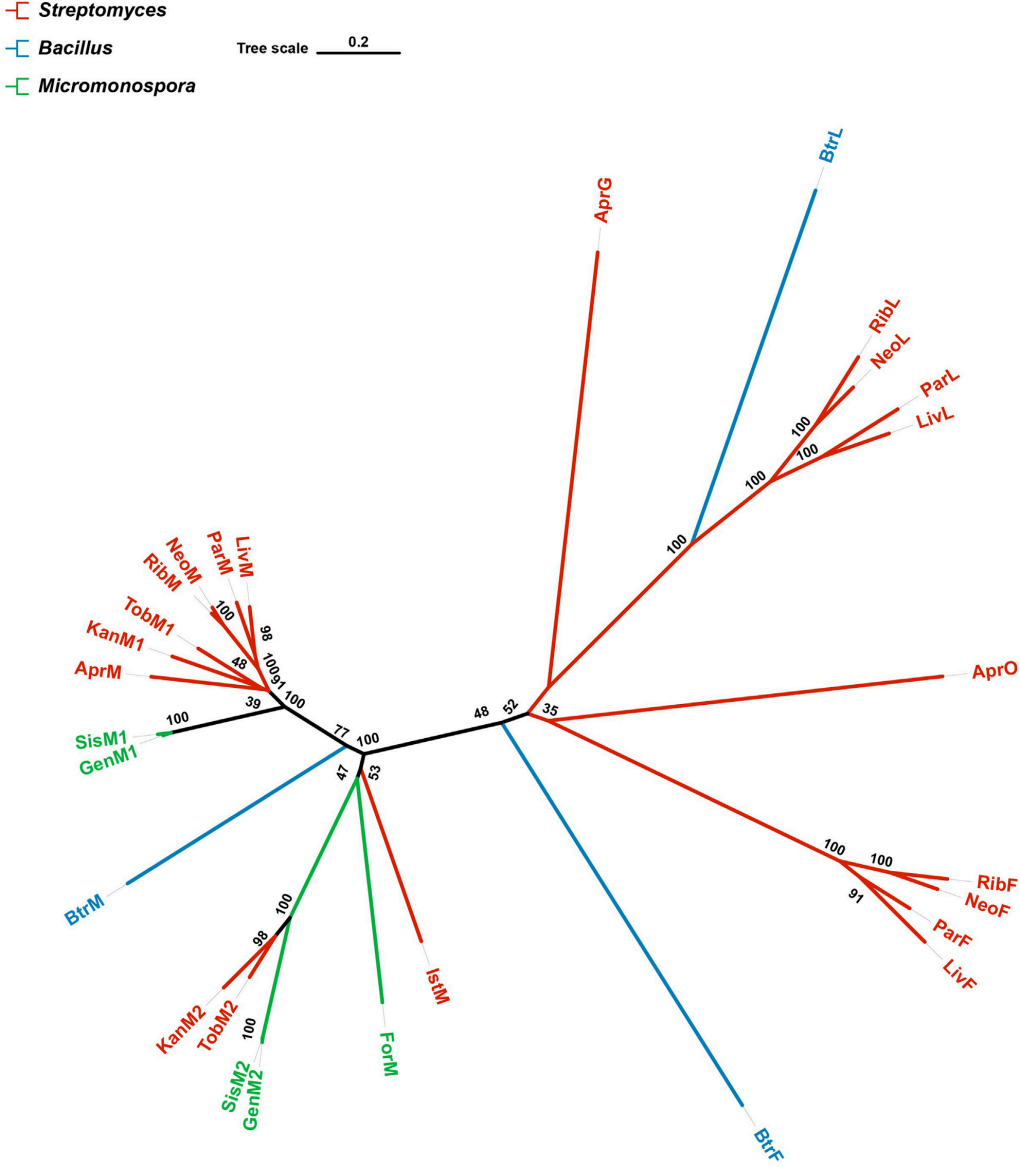
A phylogenetic tree of 28 proteins known in charge of glycosylation involved in AGs biosynthesis. The tree was constructed with the neighbor-joining method having the number of bootstrap replications set to 1,000. Branches are colored according to their source organisms.

A phylogenetic tree was constructed to analyse the 28 proteins known to be responsible for glycosylation involved in AGs biosynthesis. The tree showed that GenM2, SisM2, KanM2 and TobM2 were phylogenetically very close to each other and formed a subclade in the tree (Figure 3), consistent with the fact that all these four enzymes catalyse paromamine glycosylated at the C-6 position. Both KanM2 and GenM2 belong to the GT-4 family, with a 58% sequence identity of each other.

### 3.2 Acquisition of mutants

**FIGURE 4:**
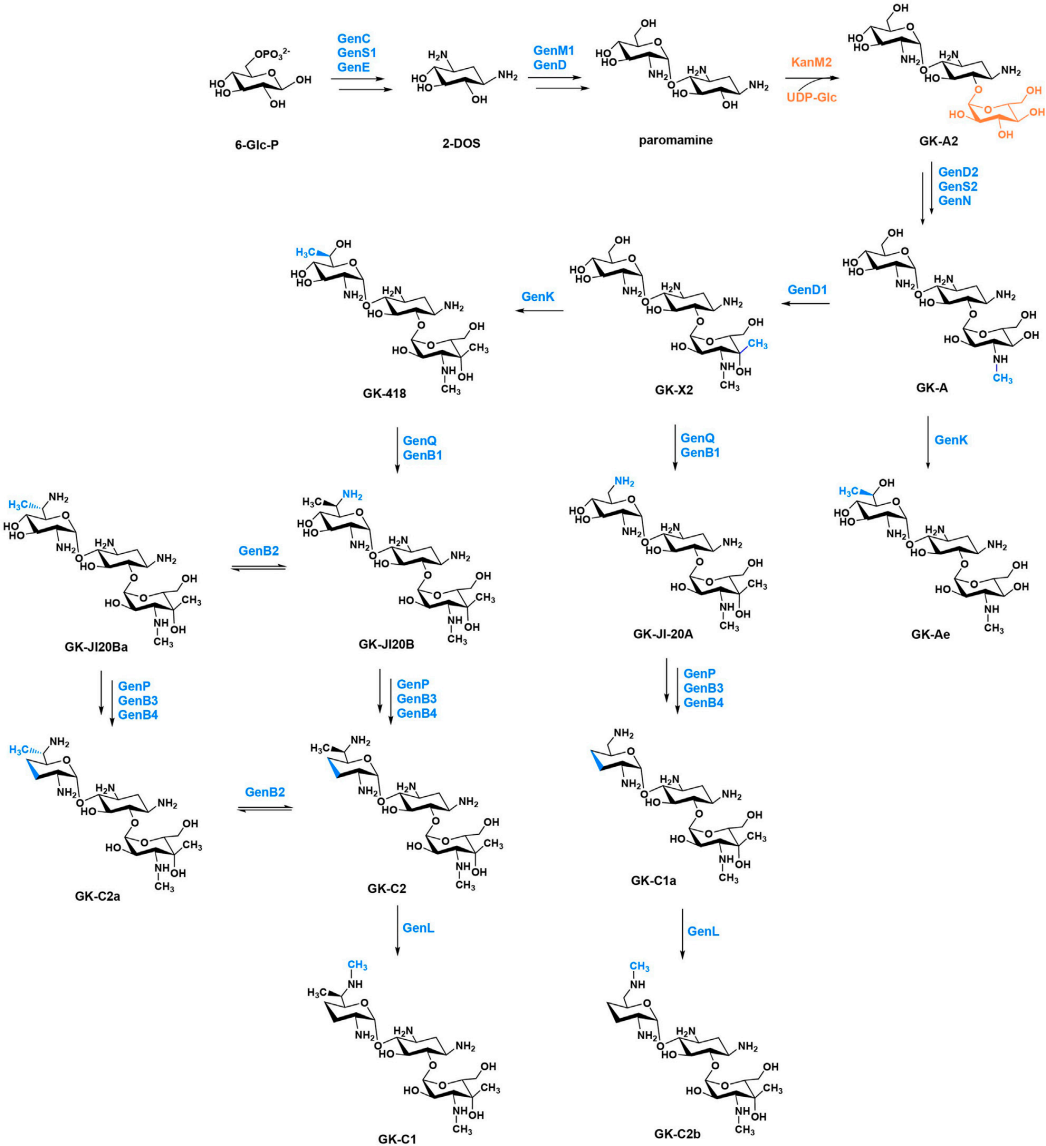
The proposed biosynthetic pathway of GKs. The functional groups and the corresponding enzymes from gentamicin and kanamycin are marked in blue and orange, respectively.

**FIGURE 5:**
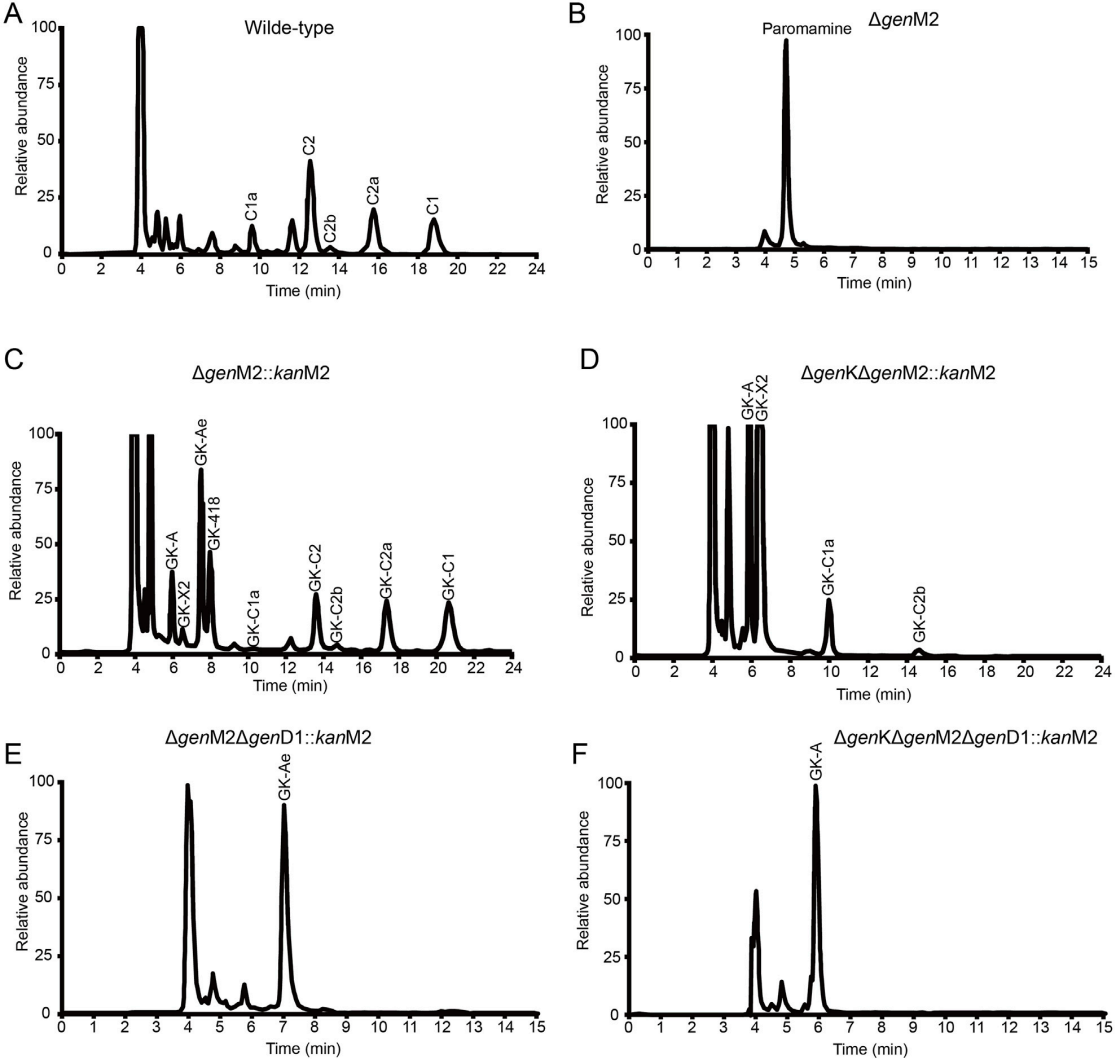
HPLC-ELSD analysis of production in wild-type and mutant strains. **(A–F)** HPLC-ELSD analysis of production in the following strains, including wild-type **(A)**, ∆*gen*M2 **(B)**, ∆*gen*M2::*kan*M2 **(C)**, ∆*gen*K∆*gen*M2::*kan*M2 **(D)**, ∆*gen*M2∆*gen*D1::*kan*M2 **(E)**, and ∆*gen*K∆*gen*M2∆*gen*D1::*kan*M2 **(F)**.

KanM2, instead of GenM2, was able to catalyse UDP-Glc attach to paromamine to produce pseudotrisaccharide in gentamicin synthesis pathway, and then modifications occurred on this pseudotrisaccharide to generate a series of gentamicin derivatives (Figure 4). We constructed the Δ*gen*M2 mutant (Supplementary Figure S2), with *gen*D1 under the control of the P*hrd*B promotor to preclude possible polar effects, which presented the abolished native pseudotrisaccharides production and accumulated paromamine in abundance as compared to the wild-type strain (Figures 5A, B). Then we constructed the *kan*M2 expression plasmid pKCM2L, where the *kan*M2 gene was under the control of the promoter *kas*Op*, and introduced it into the wild-type strain of *M. echinospora* to generate the Δ*gen*M2::*kan*M2 mutant (Supplementary Figure S2).

HPLC-ELSD and LC-ESI-HRMS analysis showed that a series of new products in the culture extract of Δ*gen*M2::*kan*M2 were successfully generated (Figure 5C). The main products of wild-type strain were gentamicin C components, while the Δ*gen*M2::*kan*M2 mutant produced more intermediates and less GK-C1a with a similar level of the rest four GK-C components under the same fermentation condition. Due to the low yields of some components and the similar structure of GKs in Δ*gen*M2::*kan*M2, the isolation and purification of mono-components was challenging. The deletion of GenK caused all products flown to the left branch (Figure 2). Based on this, we introduced pKCM2L into Δ*gen*K (a mutant strain constructed as per the protocol (Li et al., 2013)) to generate the strain Δ*gen*KΔ*gen*M2::*kan*M2 (Supplementary Figure S2). The mutant strain increased the production of GK-C1a and GK-C2b as expected, but also accumulated intermediates of GK-A and GK-X2 (Figure 5D). In this work, we constructed another *kan*M2 expression plasmid pKCM2LS2, by which the *kan*M2 gene was introduced into the wild-type and Δ*gen*K strains of *M. echinospora* to replace the *gen*M2 and *gen*D1 via the double-crossover homologous recombination, generated the Δ*gen*M2Δ*gen*D1::*kan*M2 and Δ*gen*KΔ*gen*M2Δ*gen*D1::*kan*M2 mutants (Supplementary Figure S3). As predicted, the two mutants produced a high yield of GK-Ae and GK-A respectively (Figures 5E, F, Supplementary Figure S4).

All these mutant strains were fermented to accumulated the corresponding GK products. Cultures were purified primarily via ion-exchange resins, then subjected to a HPLC-ELSD system to separate mono components. We successfully isolated GK-418, GK-C2, GK-C2a and GK-C1 from the strain Δ*gen*M2::*kan*M2 and GK-X2 and GK-C1a from the strain Δ*gen*KΔ*gen*M2::*kan*M2 with enough quantity and purity. Besides, GK-Ae and GK-A (the chemical structures of GK-A and GK-Ae in Supplementary Table S4 and Supplementary Figures S5, S6) were purified from the strains including Δ*gen*M2Δ*gen*D1::*kan*M2 and Δ*gen*KΔ*gen*M2Δ*gen*D1::*kan*M2 respectively. However, GK-C2b was not in required purity for the following structure and activity characterizations. The structural data of GK-C2a, GK-X2, GK-418, GK-C1a, GK-C2, and GK-C1 obtained here were consistent with the previous report (Jian et al., 2024) (Supplementary Table S4 and Supplementary Figure S7).

### 3.3 PTC readthrough activity of GKs compared using a dual luciferase reporter assay

To compare the PTC readthrough activity of GKs, we first assessed the ability of the eight purified GKs to suppress the R213X mutation, using a dual-luciferase reporter assay. We introduced the stop codon of the R213X and the surrounding nucleotide context (four codons upstream and three codons downstream) into this reporter vector at the start of luciferase coding phase. The reporter vector was transfected into the HEK-293 cells which were incubated with varied concentrations of the eight novel compounds for 48 h. In the assays, G418 and gentamicin were applied as the positive controls (Floquet et al., 2011). The fluc and rluc luminescence in the whole-cell lysates obtained from the AGs-treated HEK293 cell culture were measured to assess the level of PTC readthrough. The ratio of rluc to fluc luminescent signals determined in cells containing the R213X construct was normalized to the rluc/fluc value in cells containing the corresponding wild-type construct, thereby enabling the measurement of the PTC-readthrough efficiency.

**FIGURE 6:**
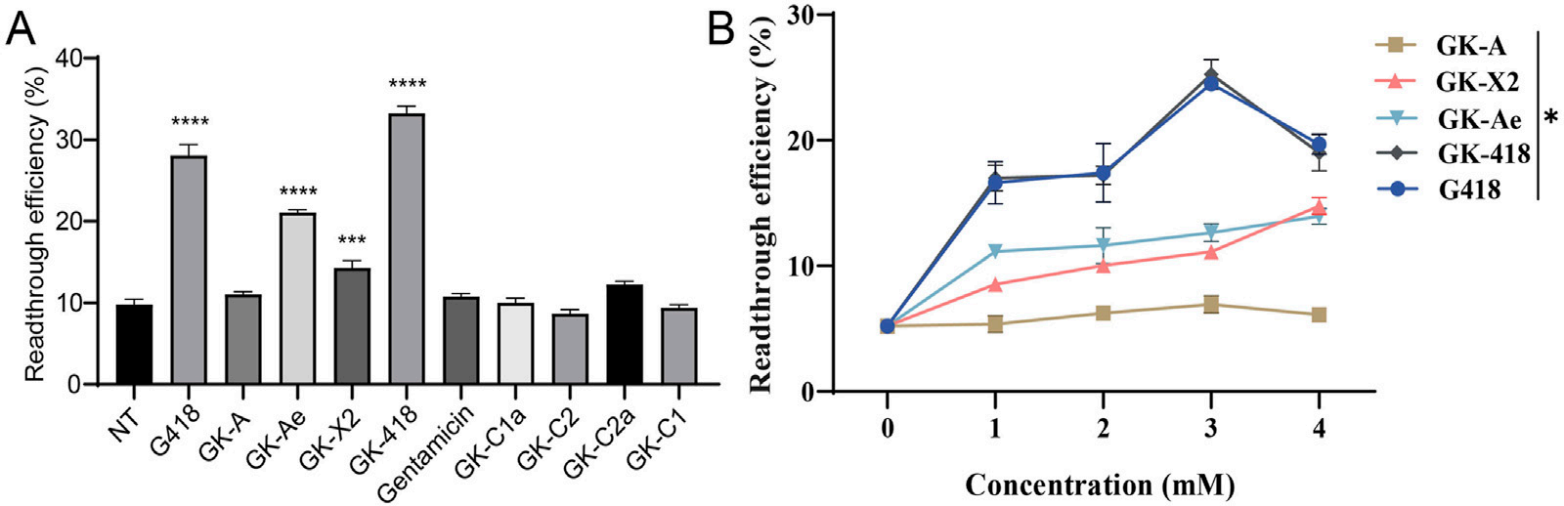
PTC readthrough activity of GKs compared using a dual luciferase reporter assay. HEK-293 cells containing a dual luciferase reporter (213X) were pre-treated with varying concentrations of compounds, and dual-luciferase reporter assay was performed on 48 h. **(A)** PTC readthrough activity of eight purified GKs at 1 mM as well as G418 and gentamicin. **(B)** Normalized PTC readthrough activity of GK-A, GK-X2, GK-Ae and GK-418 as well as G418. The data were shown as mean ± SEM (n = 4–6). Statistical analyses were performed using one-way ANOVA with Dunnett’s multiple comparisons test. * vs. NT; *p < 0.05, ***p < 0.001, ****p < 0.0001.

Based on the results, G418, GK-Ae, GK-X2, GK-418 potently stimulated PTC readthrough at the concentration of 1 mM (28.10%, 21.09%, 14.29% and 33.25% respectively), while GK-A, gentamicin and GK-C components showed little stimulation of PTC readthrough (Figure 6A). Induced readthrough efficiency of G418 was higher than that of GK-Ae and GK-X2, but lower than GK-418. Compared with gentamicin, GK-C2a showed a little higher readthrough activity, while GK-C1a, GK-C2 and GK-C1 showed lower activity. Nonetheless, no statistical significance on the induced readthrough efficiency was seen when comparing the basal readthrough efficiency of untreated cells (9.81%) with those cells treated with GK-A and GK-C components as well as gentamicin (Figure 6A, *p* > 0.05). These results suggested that gentamicin may be insensitive to this reporter system.

Further determination on the readthrough efficiency of GK-X2, GK-Ae, GK-418 and G418 displayed a rising trend with the increasing concentrations, while GK-A showed no obvious change (Figure 6B, *p* < 0.05). Moreover, GK-418 and G418 showed a reduced activity at the concentration of 4 mM.

### 3.4 The structures of GKs affected the *in-vitro* cytotoxicity

**FIGURE 7:**
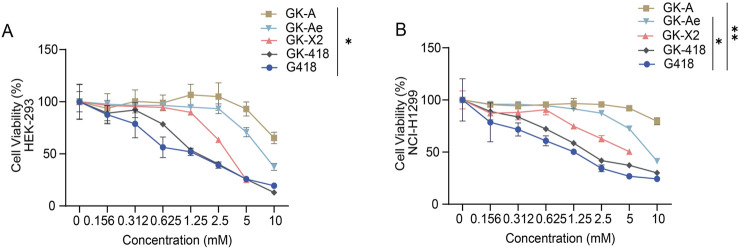
*In-vitro* toxicity assessment of GK intermediates. **(A, B)** The *in-vitro* toxicity assessment of GK intermediates to **(A)** HEK-293 and **(B)** NCI-H1299 cells was determined based on the calculated cell viability at 48 h following the treatment with different compounds in a series of concentrations. Data are represented as mean ± SEM (n = 6). Statistical analyses were performed using one-way ANOVA with Dunnett’s multiple comparisons test.

The cell toxicity of the compounds was examined according to the half-maximal lethal concentration (LC_50_) and the concentration at which 75% of the cells are viable (LC_25_) in HEK-293 cells, with G418, one of the most cytotoxic aminoglycosides, as reference. Among all the tested compounds, G418 exhibited the lowest LC_50_ value (1.31 mM). The LC_50_ values of GK-A, GK-Ae, GK-X2 and GK-418 decreased, with >10, 7.79, 3.14 and 1.72 mM, respectively. Similar trend was seen based on the results on the determination of LC_25_. Compared with that of G418, the cytotoxicity of GK-418 was slightly lower, while the cytotoxicity of other three compounds was significantly reduced (Figure 7A and Table 1). The cell viability of GK-A was significantly different from those of the control group (Figure 7A, *p* < 0.05). To better analyse their safety, the cytotoxicity of these compounds on lung adenocarcinoma cells NCI-H1299 cells was measured, and we observed a similar trend to that in HEK-293 cells (Figure 7B and Table 1). In detail, the LC_50_ and LC_25_ values of GK-A, GK-Ae, GK-X2 and GK-418 were higher than that of G418. And cell viability of GK-A and GK-Ae were significantly different from those of the control group (Figure 7B, *p* < 0.05). These results indicated that the cytotoxicity of G418 was reduced by introducing the second sugar scaffold (ring III) from garosamine to kanosamine.

**TABLE 1:** Comparative cell toxicity of G418 and GK intermediates in HEK-293 and NCI-H1299 cells.

Compound	LC_50_		LC_25_	
	HEK-293	NCI-H1299	HEK-293	NCI-H1299
GK-A	>10	>10	8.84 ± 0.31	>10
GK-Ae	7.79 ± 0.22	8.46 ± 0.16	4.55 ± 0.21	4.10 ± 0.09
GK-X2	3.14 ± 0.03	5.53± 0.48	1.94 ± 0.03	1.25 ± 0.08
GK-418	1.72 ± 0.02	2.24 ± 0.03	0.63 ± 0.04	0.49 ± 0.02
G418	1.31 ± 0.12	1.16 ± 0.09	0.32 ± 0.05	0.22 ± 0.06

### 3.5 GKs induced the expression of full-length p53 from the R213X nonsense mutant

**FIGURE 8:**
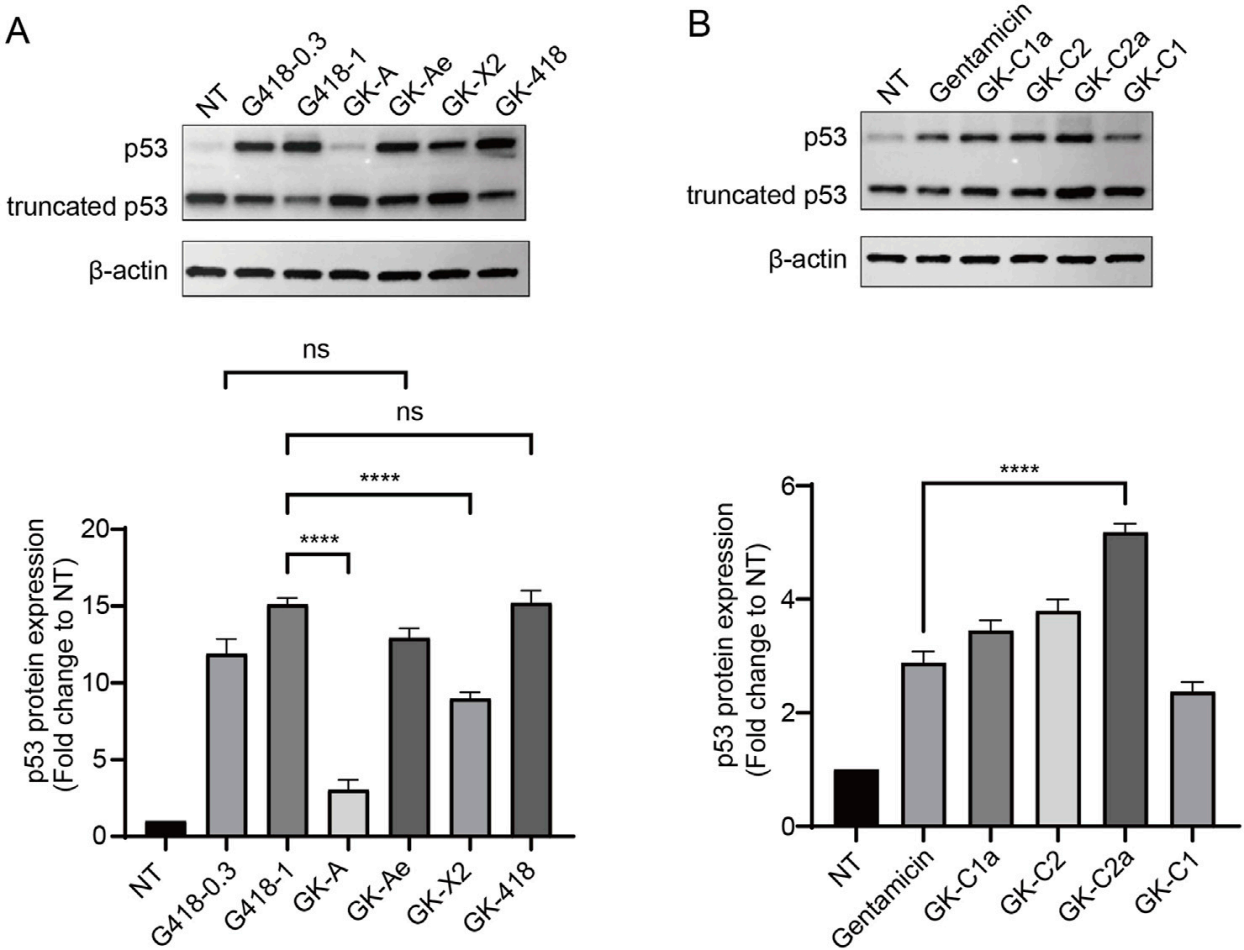
Induction of full-length p53 via GKs intermediates. **(A, B)** Western blotting analysis of 213X-transfected NCI-H1299 cells treated with test compounds of the indicated concentrations for 48 h. The relative expression of p53 was represented as the fold change compared with NT (untreated) following normalization to β-actin. Data are represented as mean ± SEM (n = 3). Statistical analyses for each panel were performed using one-way ANOVA with Dunnett’s multiple comparisons test. * vs. NT; ****p < 0.0001, ns indicates *p* > 0.05.

To further investigate the activity of derivates, we used human lung adenocarcinoma cells NCI-H1299 (p53-null) carrying R213X nonsense mutant of p53 gene as a physiological system. The production of the full-length p53 protein was tested. NCI-H1299 cells (R213X) were treated with the eight compounds at 1 mM for 48 h with G418 (0.3 and 1 mM) and gentamicin (1 mM) as positive controls (Figure 8). In NCI-H1299 cells with the absence of treatment (NT), Western blotting revealed a weak but detectable band of 53 kDa (which corresponded to the full-length p53) in these cells, which hinted that the spontaneous readthrough could generate detectable proportion of full-length protein, when the expressions of nonsense mutant genes are driven by strong promoters. In the meantime, it was revealed that GK-418 induced a higher level of full-length p53 than GK-A, GK-Ae and GK-X2, and same to G418 at concentration of 1 mM (Figure 8A). Moreover, GK-Ae induced a higher level of full-length p53 than that of GK-X2, and GK-A produced the least efficiency on inducing the protein expression of p53 (Figure 8A). Additionally, a higher level of full-length p53 was seen in the cells treated with 1 mM G418 than those cells treated with 0.3 mM of G418 (the maximum dose compatible with 75% cell viability) (Figure 8A), and gentamicin, despite its less efficiency, also induced the full-length p53. As shown in Figure 8B, the efficiency of all derivatives, except GK-C1, in inducing the p53 level was higher than gentamicin, with GK-C2a manifesting the highest efficiency (Figure 8B).

**FIGURE 9:**
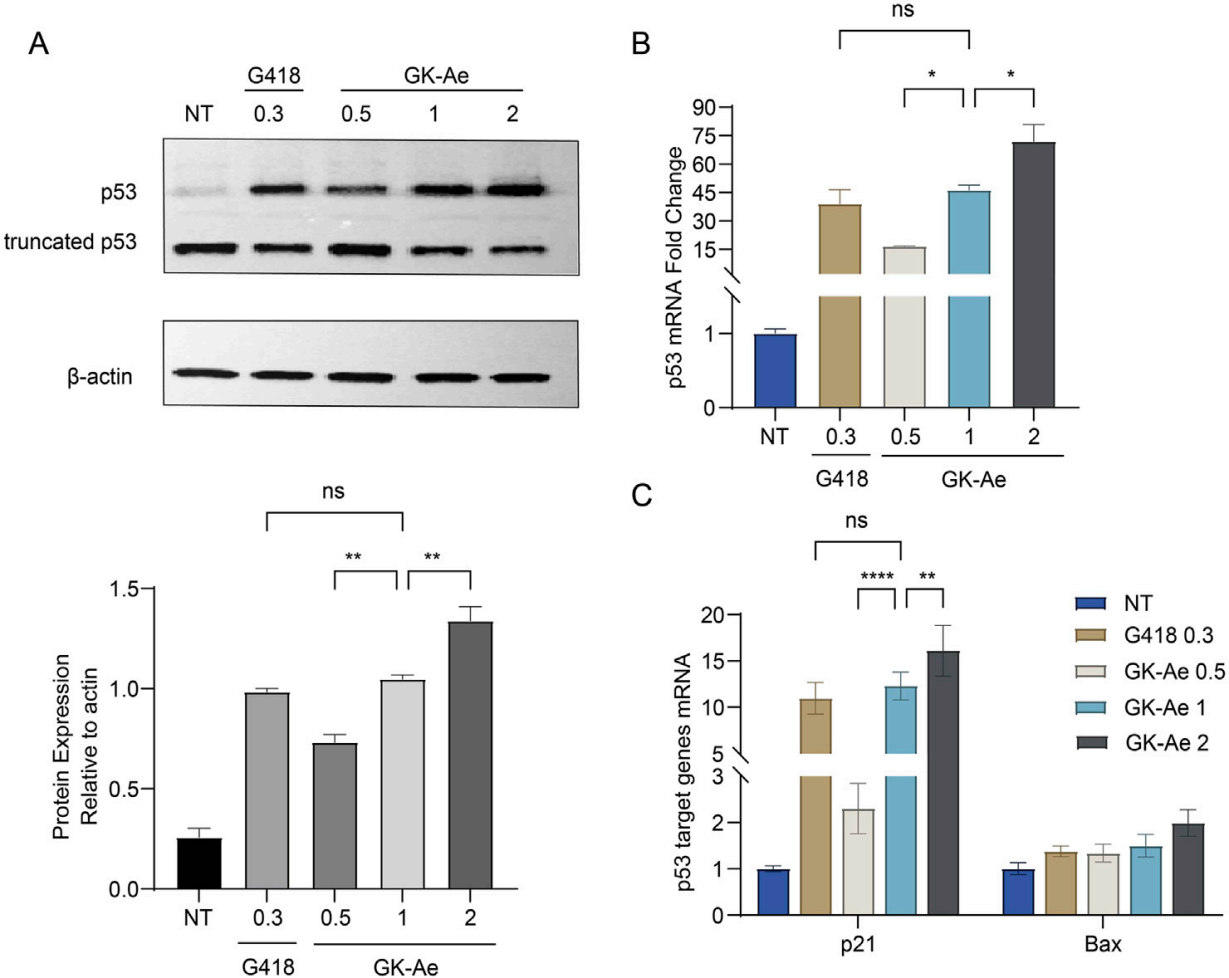
GK-Ae restores production of the full-length p53 protein and stabilises the mutant p53 mRNA. NCI-H1299 cells carrying the R213X nonsense mutation were treated with GK-Ae (0.5, 1, 2 mM) or G418 (0.3 mM) for 48 h. **(A)** Western blotting assay showing the protein band of p53 following the treatment of GK-Ae or G418. **(B)** Relative p53 mRNA level in NCI-H1299 cells of each group was determined by qRT-qPCR. **(C)** Relative mRNA levels of p53 target genes p21 and Bax in NCI-H1299 cells of each group. Data are represented as mean ± SEM (n = 3). Statistical analyses for each panel were performed comparing all conditions to each other using one-way ANOVA or two-way ANOVA with Tukey’s multiple comparisons test. * vs. NT; *p < 0.05, **p < 0.01, ***p < 0.001, ****p < 0.0001. ns indicates *p* > 0.05.

Further analysis showed that GK-Ae treatment induced the production of full-length p53 protein in a dose-dependent manner (Figure 9A), proportional to the effect of the drug on readthrough level. The PTC readthrough level induced by GK-Ae at the middle dose (1 mM) was comparable to that induced by G418 at a maximum non-toxic dose (0.3 mM).

### 3.6 GK-Ae treatment stabilised mutant p53 mRNA

To determine whether GK-Ae can stabilise mRNAs, NCI-H1299 cells were transiently transfected with a p53 R213X construct. This mutation was detected at exon 6 and produced a premature UGA stop codon 50 nt upstream from the last exon–exon junction. The generated mRNA molecule was therefore considered as a canonical target for the degradation by nonsense-mediated mRNA decay (NMD) pathway. qRT-PCR was performed on NCI-H1299-213X cells which were treated by various concentrations of GK-Ae for 48 h to determine mRNA levels, and those cells treated with G418 served as a positive control. It was found that GK-Ae induced the mRNA levels of p53 in a dose-dependent manner, increased from 16- to 72-fold. GK-Ae treatment at 1 mM resulted in a 46-fold induction of the mRNA levels of p53, and G418 had a similar inductor effect, which induced the 39-fold elevation of p53 mRNA level at the maximum concentration (0.3 mM) (Figure 9B).

### 3.7 GK-Ae induced mRNA expression of p21 and Bax, the two main targets of p53

To examine whether the full-length p53 protein induced by GK-Ae in NCI-H1299 cells is transcriptionally active, qRT-PCR was carried out to assess differences in the expression of the p21 and Bax genes in NCI-H1299 (213X) cells with/out GK-Ae treatment.

In each experiment, the results obtained are expressed relative to untreated cells (normalized to 1). A robust dose-dependent induction of p21 mRNA level was observed in the GK-Ae-induced NCI-H1299 (213X) cells (Figure 9C), which was consistent with the fact that GK-Ae induced significant level of full-length p53 (Figure 9A). The mRNA level of Bax was also upregulated in a dose-dependent manner, but this effect was not statistically significant. The induced levels of p21 and Bax mRNA by 1 mM GK-Ae were 12.3 and 1.5 times, respectively, while the values by 0.3 mM G418 were 11 and 1.4 times, showing an equivalent activity. These results collectively demonstrated that GK-Ae-induced full-length p53 protein exerted a partial transcriptional effect.

### 3.8 GK-Ae induced the translocation of p53 protein to the nucleus

The p53 protein is synthesized in the cytoplasm and transported to the nucleus, and the truncated p53 localized in the cytoplasm is nonfunctional. Since the N-terminal p53 antibody we used cannot differentiate between full-length p53 and C-terminally truncated p53 (data not shown), immunofluorescence assay was performed using PAb421, an antibody that specifically recognized the p53 C-terminus, to investigate the subcellular localization of the full-length p53 protein generated by readthrough in the NCI-H1299 cells transfected with p53 213X construct.

**FIGURE 10:**
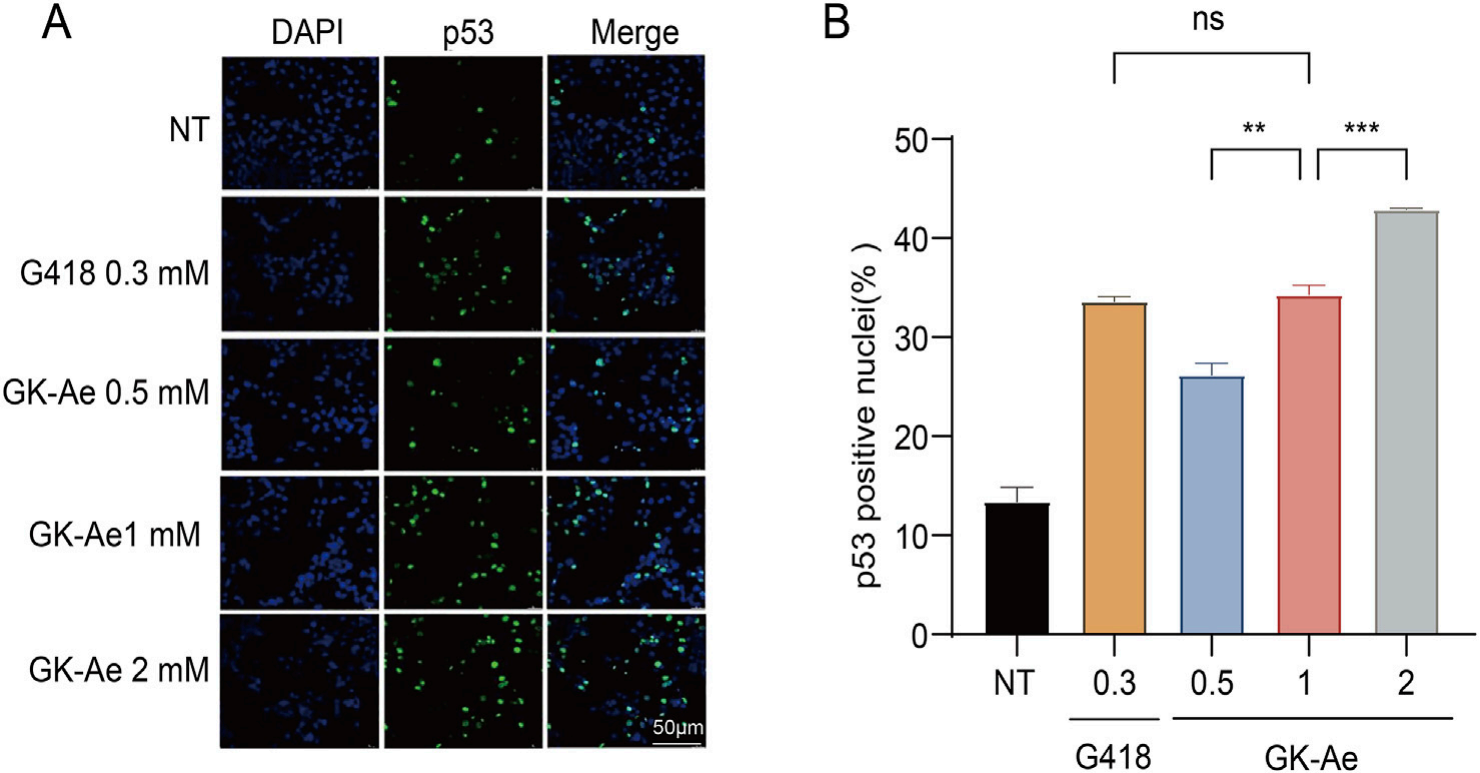
Nuclear localization of induced full-length p53 protein. **(A, B)** Immunofluorescence assay determining the fluorescence activity of p53 in 213X-transfected H1299 cells treated with/out GK-Ae (0.5, 1, 2 mM) and G418 (0.3 mM) for 48 h. Quantified data are represented as mean ± SEM (n = 3). Statistical analyses for each panel were performed using repeated measures one-way ANOVA with Tukey’s multiple comparisons test. * vs. NT; **p < 0.01, ***p < 0.001. ns indicates *p* > 0.05.

Based on the results, a weak nuclear p53 signal was detected in untreated cells, which expressed the basic readthrough (Figure 10A). After 48-h treatment with GK-Ae (0.5, 1, and 2 mM), the percentage of cells containing nuclear p53 was dose-dependently increased (from 26.15% to 42.83%) compared to untreated cells, indicating that the induced p53 protein was properly transported into the nucleus. Moreover, there was no statistical difference with regards to the p53 fluorescence intensity in cells following the treatment of G418 at 0.3 mM and GK-Ae at 1 mM (*p* > 0.5, Figure 10B).

### 3.9 Re-expressed p53 increased cancer cell apoptosis

**FIGURE 11:**
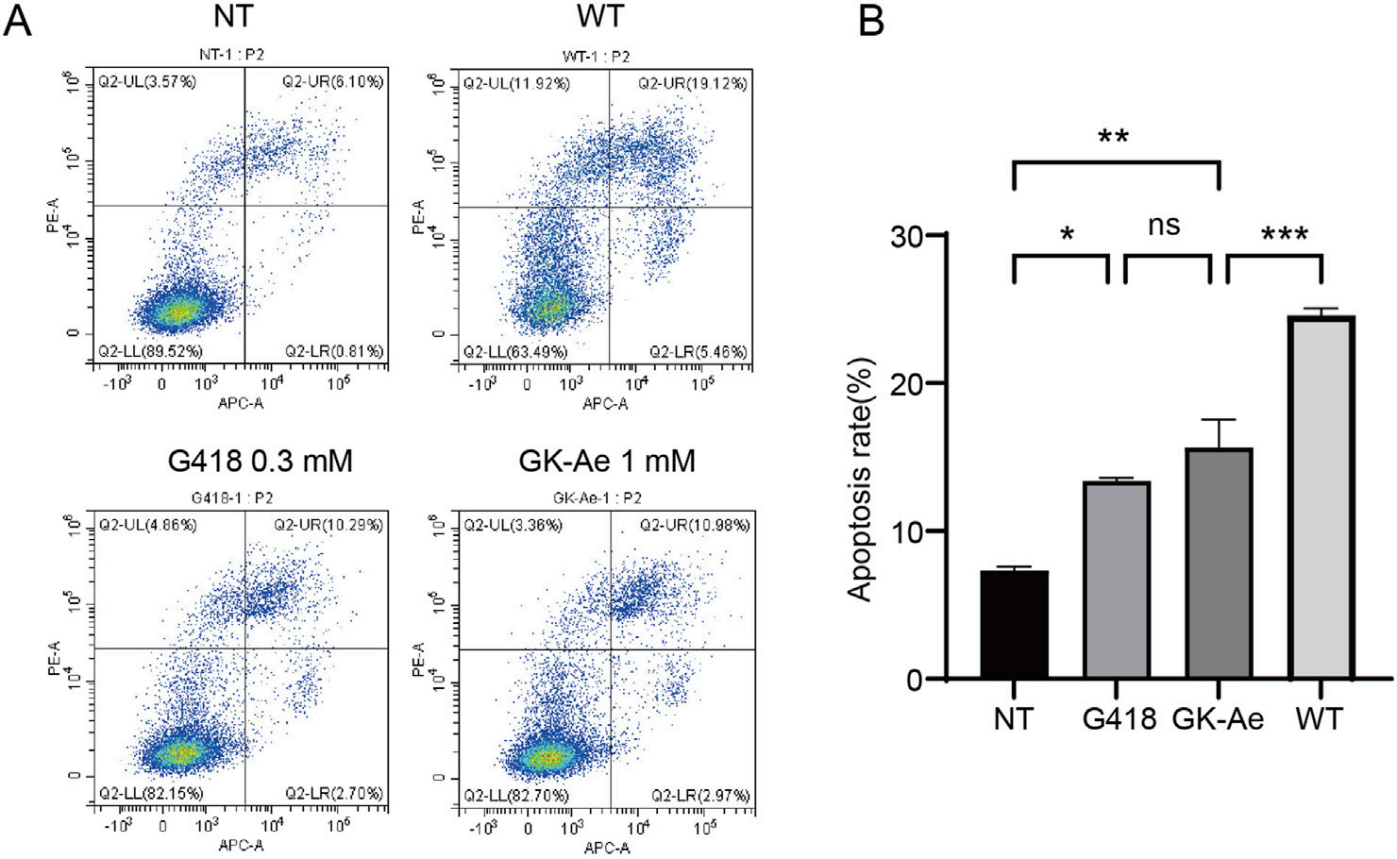
GK-Ae induces R213X p53-dependent apoptosis in cancer cells. **(A, B)** Flow cytometry was used to determine the apoptosis of H1299-R213X cells with/out the treatment with 1 mM GK-Ae or 0.3 mM G418 for 48 h. Data are represented as mean ± SEM (n = 3). Statistical analyses were performed using one-way ANOVA with Tukey’s multiple comparisons test. * vs. NT; *p < 0.05, **p < 0.01, ***p < 0.001. ns indicates *p* > 0.05.

Since p53 initiates cell apoptosis, we then investigated whether the full-length p53 protein induced by GK-Ae treatment affected the apoptosis in cancer cells. Accordingly, p53-R213X construct was transiently transfected into NCI-H1299 cells to examine the p53-mediated apoptosis (Chen et al., 1996). In the absence of treatment, NCI-H1299-p53R213X cells showed a basal percentage of apoptosis (7.34%) (Figures 11A, B). After 48 h of treatment, the apoptosis rate of NCI-H1299-p53R213X cells induced by 1 mM GK-Ae was similar to the cells treated with 0.3 mM G418 (15.63% and 13.39% respectively), whilst being lower than those cells transfected with p53-WT (24.58%) (Figures 11A, B). These results suggested that the treatment with GK-Ae increased the apoptosis in NCI-H1299 cells containing p53 R213X, indicating a p53-dependent apoptotic effect.

## 4 Discussion

Nonsense mutations, which introduce a PTC into the protein-coding gene sequence, account for around 11% of all known gene lesions leading to human genetic diseases (Li et al., 2023). AG antibiotics are an important class of readthrough inducers, but the side effects after long-term use limit their clinical application (Pokrovskaya et al., 2010).

The gentamicin biosynthetic pathway has extensive substrate tolerance and complex modification system, which can be used to prepare new AG analogues. C6′-hydroxy and C6′-methyl groups of gentamicin are important for the readthrough activity of PTC (Kandasamy et al., 2012; Prokhorova et al., 2017). Towards this end, plasmids encoding KanM2 were introduced into the wild-type and mutant strains of *M. echinospora* ATCC15835 by homologous recombination. With the application of this combinatorial biosynthetic approach, eight novel gentamicin derivatives were obtained, in which xylose was replaced with glucose in the ring III scaffold. The antimicrobial activity of genkamicins was tested by Jian et al. (Jian et al., 2024). We investigated the relationship between the biological activities and structures, including PTC readthrough activity and cytotoxicity of the genkamicins. We firstly explored the ability of GKs to promote readthrough of nonsense mutation using a dual-luciferase reporter assay, and then verified whether they could restore of full-length p53 protein by Western blotting. The eight compounds showed different levels of readthrough activity. The readthrough activity of G418 and GK-418 was basically the same, indicating the variation of ring III from xylose to glucose had no impact. Differences in GK-A, GK-X2, GK-Ae, and GK-418 suggested the methyl modification improved the readthrough activity of compounds, with C6′-methyl being more efficient than C4″-methyl. The higher activity of GK-418 compared to GK-C2 proved that the C6′-hydroxy and C3′,4′-dihydroxy moieties are crucial for readthrough activity, which was in agreement with previous reports (Kandasamy et al., 2012; Lee et al., 2023). Additionally, the differences in cytotoxicity between the intermediates and G418 demonstrated the increased toxicity of methyl groups, with C4′-methyl having a greater effect than C6′-methyl. The only structural difference between G418 and GK-418 was that the ring III scaffold was replaced by xylose with glucose, suggesting that glucose as ring III contributed to a lower cytotoxicity. Collectively, it demonstrative that C3′,4′-dihydroxy, C6′-hydroxy, C6′-methyl and C4″-methyl enhanced the readthrough activity. Meanwhile, C6′-methyl, C4″-methyl and the transformation of ring III from glucose to xylose increased cytotoxicity. The cytotoxicity of GK-C components was not examined due to their low readthrough activity. GK-C2a showed more efficient at PTC readthrough than other GK-C components and gentamicin, which, in combination with data on its ototoxicity (Jian et al., 2024), suggested that (*S*)-C6′-methyl significantly increased readthrough activity and reduced toxicity compared to (*R*)-C6′-methyl.

NMD is a highly conserved quality control mechanism of cellular mRNAs that selectively degrades mRNA containing PTCs, thereby preventing the accumulation of truncated proteins that might be deleterious or non-functional (Lejeune, 2022; Supek et al., 2021). NMD can differentiate between PTC and normal termination codon (NTC) according to the existence of EJC, which located 20–24 nt upstream of each exon-exon junction. During the pioneer round of translation until the NTC, the ribosome removed all EJCs carried by the mRNA. However, its progression is disrupted and the removal of EJCs ceased if ribosome encounters a PTC located 50–55 nt upstream of an exon-exon junction. The remaining EJC signals the existence of PTC and recruits NMD factors to degrade abnormal mRNA, thereby preventing the production of truncated and potentially toxic proteins. The degradation of PTC-containing mRNAs by NMD results in the lack of transcripts available for protein synthesis, significantly reducing the efficacy of readthrough. Considering the higher level of PTC suppression and lower cytotoxicity, we selected GK-Ae for further study and characterized its effect on cancer cells. An existing study has demonstrated that AGs can induce the translational readthrough and the expression level of full-length p53 (Zhang et al., 2017). Additionally, based on the discoveries in our current study, GK-Ae induced a dose-response production of full-length p53 and stabilised mutant p53 mRNA. Then, the increased mRNA levels of target genes p21 and Bax, proportion of p53 localized in the nucleus, and apoptosis rate proved that GK-Ae could partially restore the biological activity of p53. When the level of induced full-length p53 was comparable, the LC_25_ of GK-Ae in NCI-H1299 cells was about 20 folds that of G418, which indicated that the potency ratio of GK-Ae was approximately 20-fold than that of G418 (Table 1). Although there was no direct data, based on structure-activity relationship, GK-Ae may be more effective than gentamicin Ae, as well as gentamicin X2, which have been reported to have a larger read through/safety window than G418 (Friesen et al., 2018). Thus, our data supported the feasibility of rational modification of AGs to obtain novel derivatives with higher PTC readthrough activity and lower toxicity, which could be used as drugs to treat nonsense mutations. Besides, the activities of these new analogues could be further enhanced by installing (*S*)-4-amino-2-hydroxybutanyl (AHB) group (which has been shown to improve readthrough activity and reduce toxicity (Kandasamy et al., 2012)) in the 1-N position. Such claim awaits to be validated in the future.

Although our results are promising, there are some inevitable shortcomings in our *in-vitro* assays. Since the level of readthrough relies on different factors, each assay should be adjusted to specific disease backgrounds. Further studies, including evaluation of other nonsense mutations and *in-vivo* approaches, are critical to consider potential of each compound for clinical applications. The PTC readthrough efficiency, toxicity and bioavailability of induced compounds are important issues that need to be addressed.

## 5 Conclusion

In this study, we introduced KanM2 into the biosynthetic pathway of gentamicin to produced eight derivates. Meanwhile, four engineered mutants were developed for the targeted accumulation of GK components. Of these mutants, Δ*gen*M2Δ*gen*D1::*kan*M2 and Δ*gen*KΔ*gen*M2Δ*gen*D1::*kan*M2 were used for the production of GK-Ae and GK-A. All derivates could induce the readthrough of nonsense mutation, albeit with different efficacies. Notably, GK-C2a exhibited appreciably higher PTC readthrough efficiency than other GK-C components and gentamicin. Furthermore, GK-Ae exhibited similar activity to that of G418, while possessing a significantly lower cell toxicity than that of G418.

### 5.1 Scope statement

Eight gentamicin derivatives were obtained and their nonsense mutation readthrough activity and cytotoxicity were investigated. GK-Ae displayed similar PTC readthrough activity but reduced toxicity than natural aminoglycoside G418, which is reported to have the strongest PTC readthrough activity. Moreover, GK-Ae increased the levels of both p53 and its downstream targets p21 and BAX and enhanced the apoptosis of cancer cells. These results demonstrated the potential of combinatorial biosynthesis to increase the diversification of AGs structures and provide directions for the development of new AGs with low toxicity and high PTC readthrough activity.

## Data Availability

The original contributions presented in the study are included in the article/Supplementary Material, further inquiries can be directed to the corresponding authors.

## Abbreviations

PTC, premature termination codon; AGs, Aminoglycosides; 2-DOS, 2-deoxystreptamine; HPLC, high-performance liquid chromatography; MS, mass spectrometry; NMR, nuclear magnetic resonance; ELSD, evaporative light scattering detection; HEK293, Human embryonic kidney 293; DMEM, Dulbecco’s modified Eagle’s medium; FBS, foetal bovine serum; WT, wild-type; CCK-8, Cell Counting Kit-8; OD, optical density; SDS-PAGE, SDS polyacrylamide gel electrophoresis; PVDF, polyvinylidene fluoride; PBST, PBS containing 0.1% Tween 20; HRP, horseradish peroxide; ECL, enhanced chemiluminescence; qRT-PCR, quantitative real-time PCR; DAPI, 4′,6-diamidino-2-phenylindole; SEM, standard error of the mean; GT, glycosyltransferase; GKs, genkamicins; LC_50_, half-maximal lethal concentration; LC_25_, the concentration at which 75% of the cells are viable; EJCs, exon junction complexes; NMD, nonsense-mediated decay; NTC, normal termination codon; AHB, (*S*)-4-amino-2-hydroxybutanyl.
