# Supplementary material for: Combinatorial biosynthesis of novel gentamicin derivatives with nonsense mutation readthrough activity and low cytotoxicity
Source: Front Pharmacol. 2025 Apr 24;16:1575840. doi: 10.3389/fphar.2025.1575840 (PMC12059486; doi:10.3389/fphar.2025.1575840)
Supplement: Supplementary file 1 [file Image5.pdf]

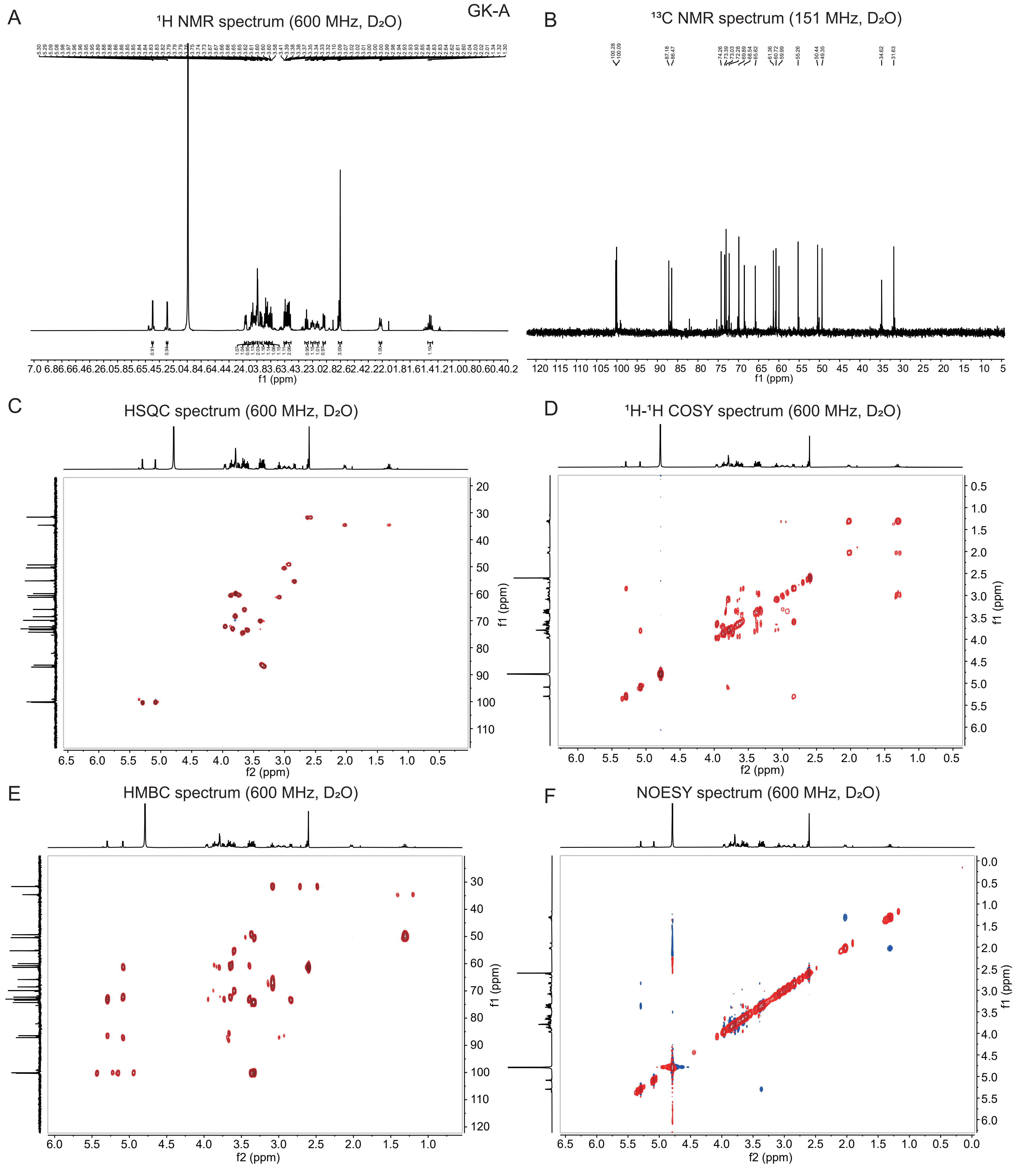
A
¹H NMR spectrum (600 MHz, D₂O)
GK-A
f1 (ppm)
B
¹³C NMR spectrum (151 MHz, D₂O)
f1 (ppm)
C
HSQC spectrum (600 MHz, D₂O)
f2 (ppm)
f1 (ppm)
D
¹H-¹H COSY spectrum (600 MHz, D₂O)
f2 (ppm)
f1 (ppm)
E
HMBC spectrum (600 MHz, D₂O)
f2 (ppm)
f1 (ppm)
F
NOESY spectrum (600 MHz, D₂O)
f2 (ppm)
f1 (ppm)
