# Supplementary material for: Combinatorial biosynthesis of novel gentamicin derivatives with nonsense mutation readthrough activity and low cytotoxicity
Source: Front Pharmacol. 2025 Apr 24;16:1575840. doi: 10.3389/fphar.2025.1575840 (PMC12059486; doi:10.3389/fphar.2025.1575840)
Supplement: Supplementary file 2 [file Table1.docx]

Supplementary Table 1. List of enzymes in charge of glycosylation involved in AGs biosynthesis.

| Enzyme | Protein ID | Strain | GenBank No. |
| --- | --- | --- | --- |
| GenM2 | CAF31433.1 | *Micromonospora echinospora* | AJ628149 |
| GenM1 | CAF31428.1 |  |  |
| KanM2 | BAD20762.1 | *Streptomyces kanamyceticus* | AB164642 |
| KanM1 | BAD20768.1 |  |  |
| TobM2 | CAH18558.1 | *Streptomyces* sp. DSM 40477 | AJ810851 |
| TobM1 | CAH18562.1 |  |  |
| SisM2 | ACN38347.1 | *Micromonospora inyonensis* | FJ160413 |
| SisM1 | ACN38342.1 |  |  |
| NeoL | CAF33322.1 | *Streptomyces fradiae* DSM 40063 | AJ629247 |
| NeoF | CAF33320.1 |  |  |
| NeoM | CAF33313.1 |  |  |
| ParL | CAF32384.1 | *Streptomyces rimosus* subsp. paromomycinus  NRRL 2455 | AJ628955 |
| ParF | CAF32382.1 |  |  |
| ParM | CAF32375.1 |  |  |
| BtrL | BAE07064.1 | *Bacillus circulans* | AB097196 |
| BtrF | BAE07070.1 |  |  |
| BtrM | BAE07063.1 |  |  |
| LivL | CAG38706.1 | *Streptomyces lividus* | AJ748832 |
| LivF | CAG38704.1 |  |  |
| LivM | CAG38698.1 |  |  |
| RibL | CAG34027.1 | *Streptomyces ribosidificus* | AJ744850 |
| RibF | CAG34029.1 |  |  |
| RibM | CAG34036.1 |  |  |
| ForM | CAF31539.1 | *Micromonospora olivasterospora* DSM 43868 | AJ628421 |
| IstM | CAI59979.1 | *Streptomyces tenjimariensis* | AJ845083 |
| AprG | CAF33041.1 | *Streptomyces tenebrarius* | AJ629123 |
| AprO | CAF33038.1 |  |  |
| AprM | CAF33046.1 |  |  |
