# Supplementary figures and images for: Combinatorial biosynthesis of novel gentamicin derivatives with nonsense mutation readthrough activity and low cytotoxicity
Source: Front Pharmacol. 2025 Apr 24;16:1575840. doi: 10.3389/fphar.2025.1575840 (PMC12059486; doi:10.3389/fphar.2025.1575840)

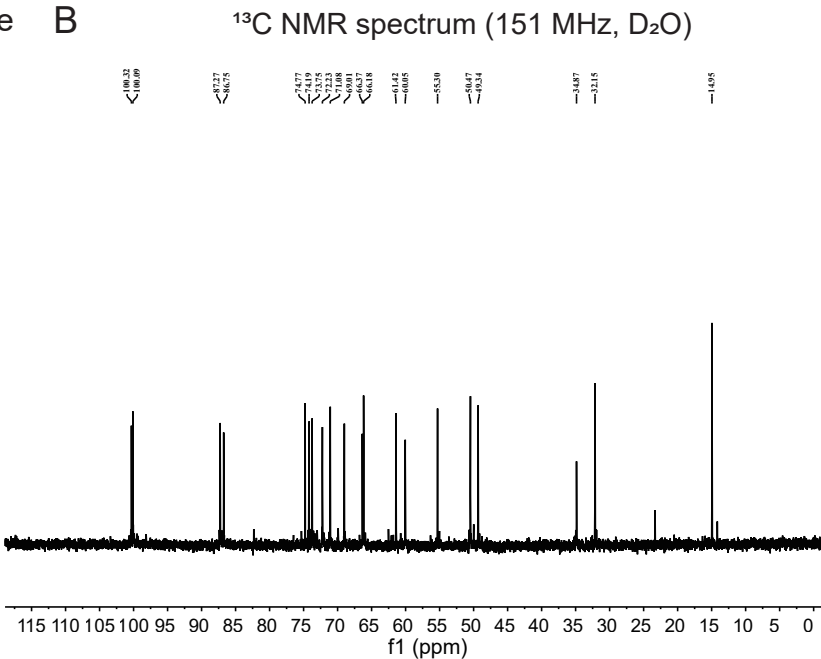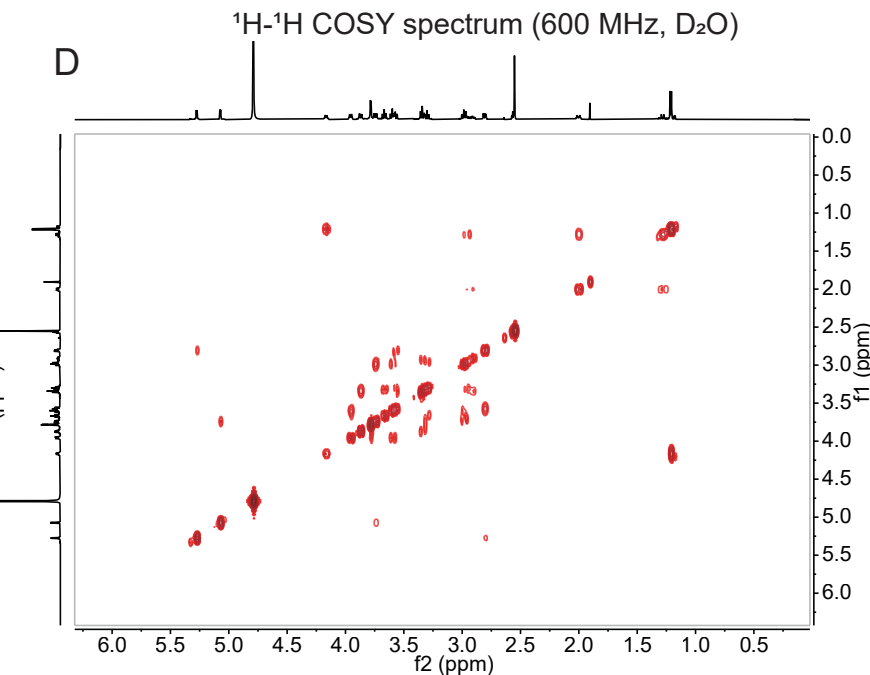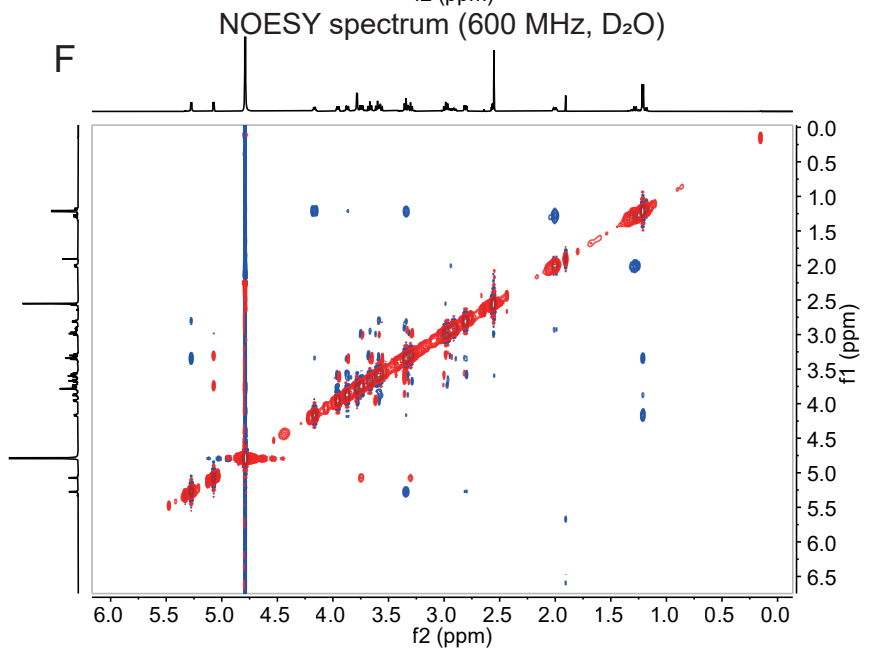

Supplement: Supplementary file 3 [file Image6.pdf]

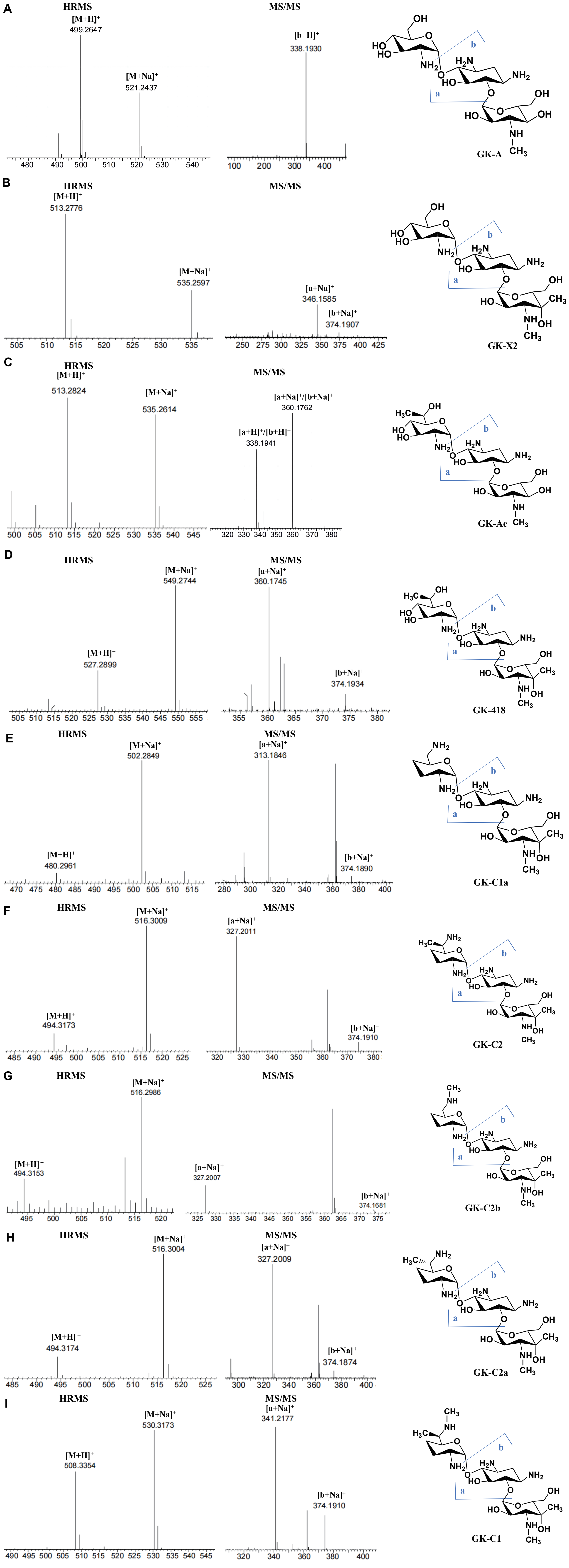

Supplement: Supplementary file 4 [file Image4.pdf]

# A

## (1) $\Delta genM2 \Delta genD1::kanM2$

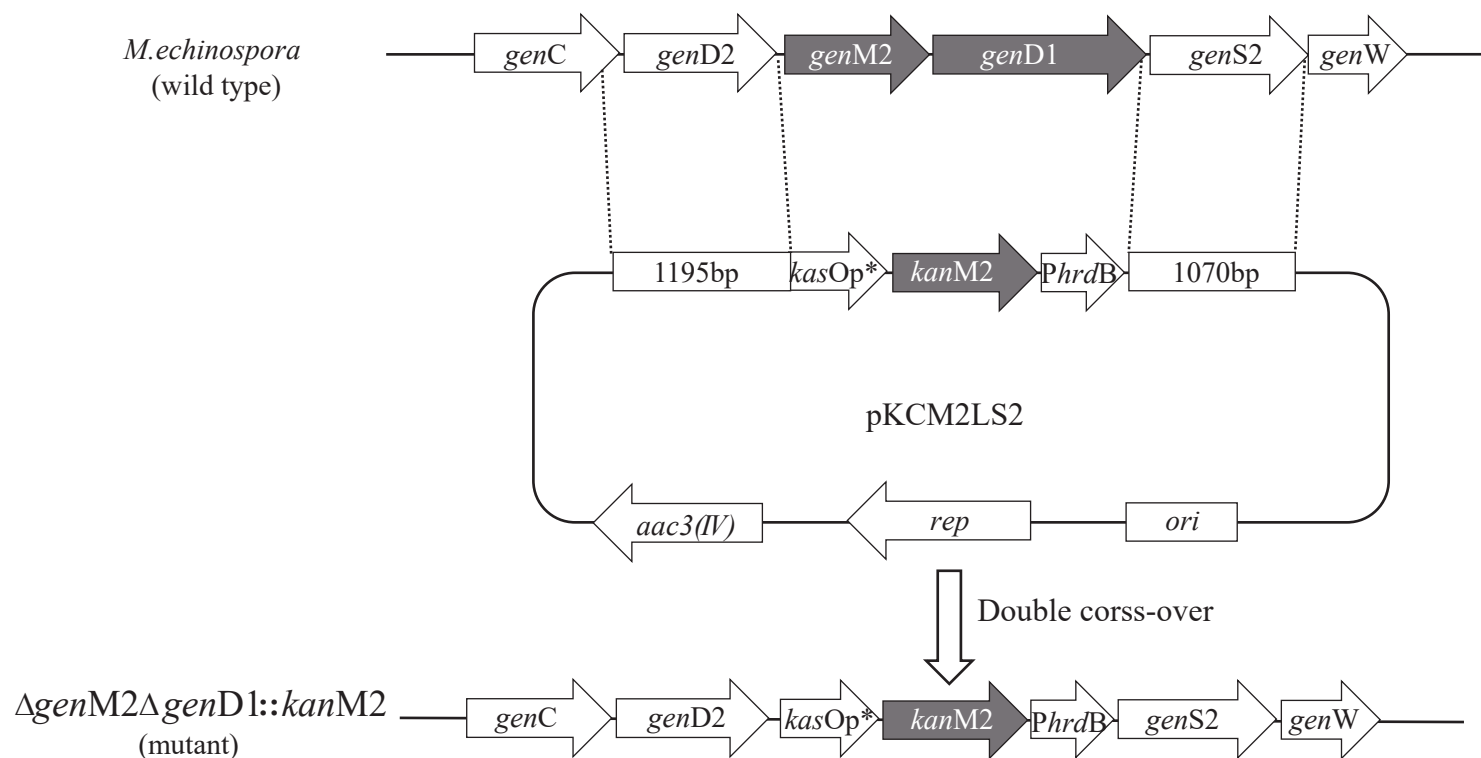

# B

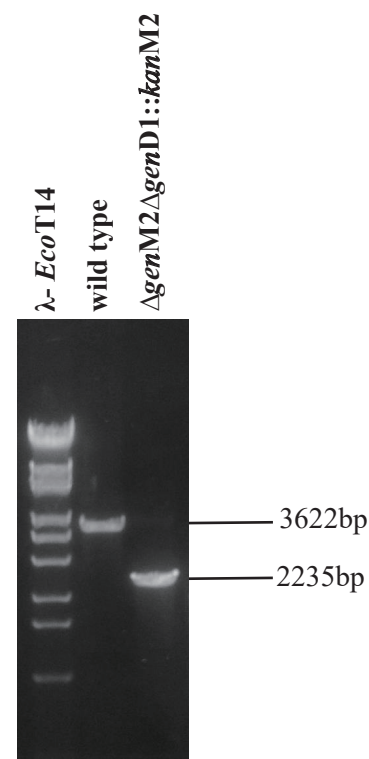

## (2) $\Delta genK \Delta genM2 \Delta genD1::kanM2$

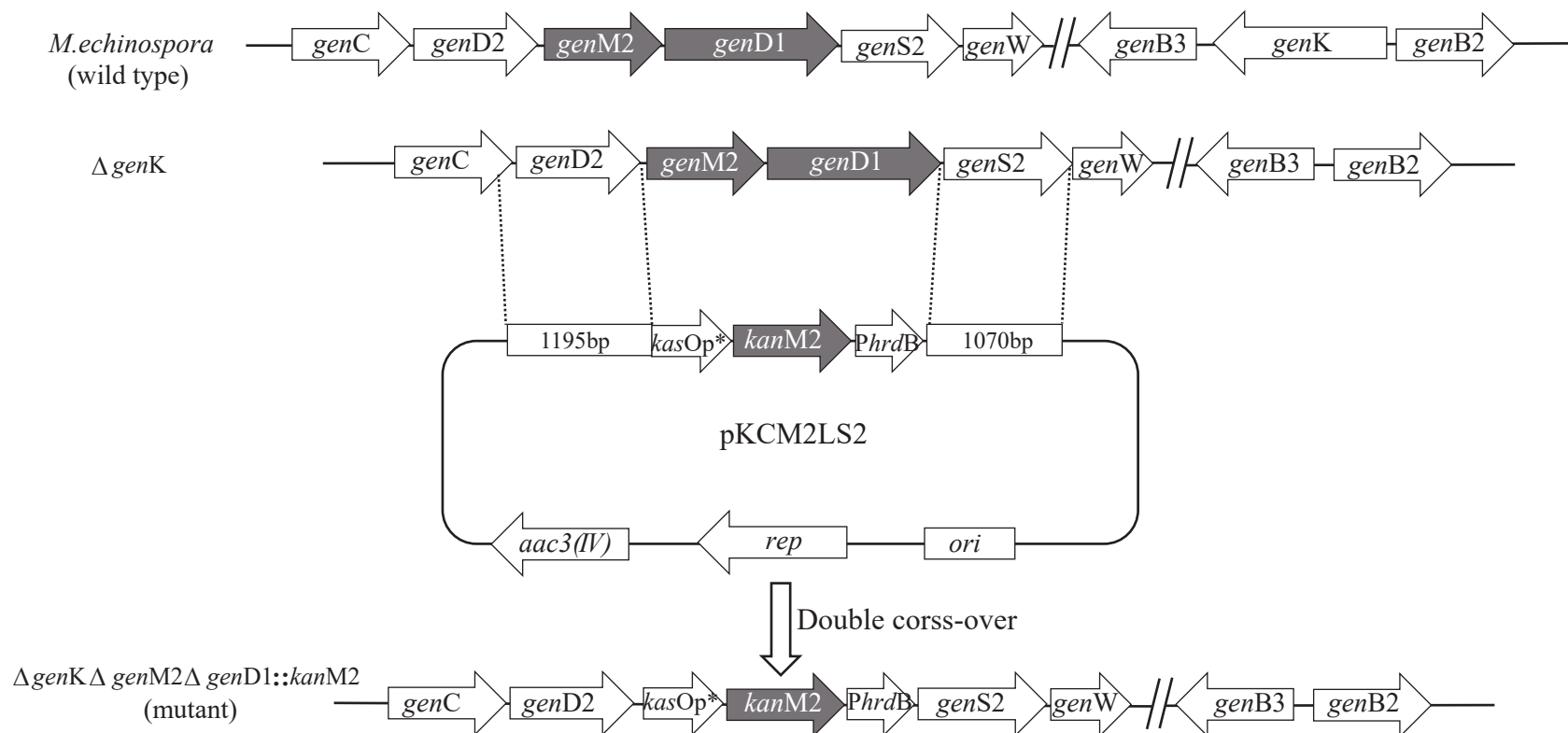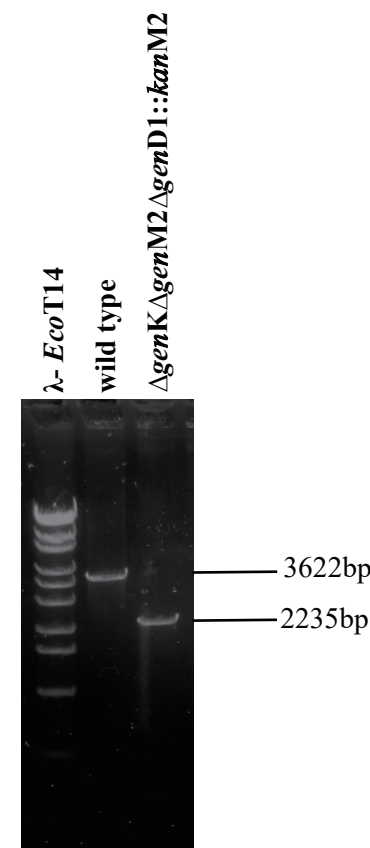

Supplement: Supplementary file 6 [file Image3.pdf]

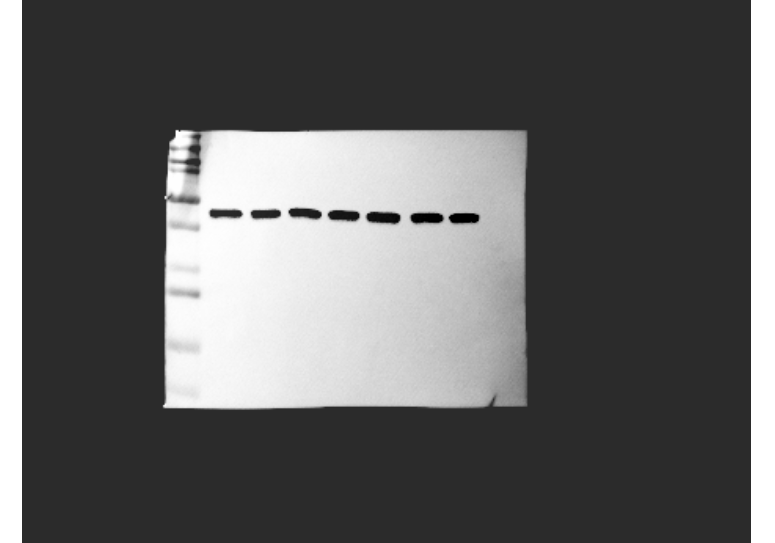

Supplement: Supplementary file 9 [file DataSheet2.zip › actin 1.tif]

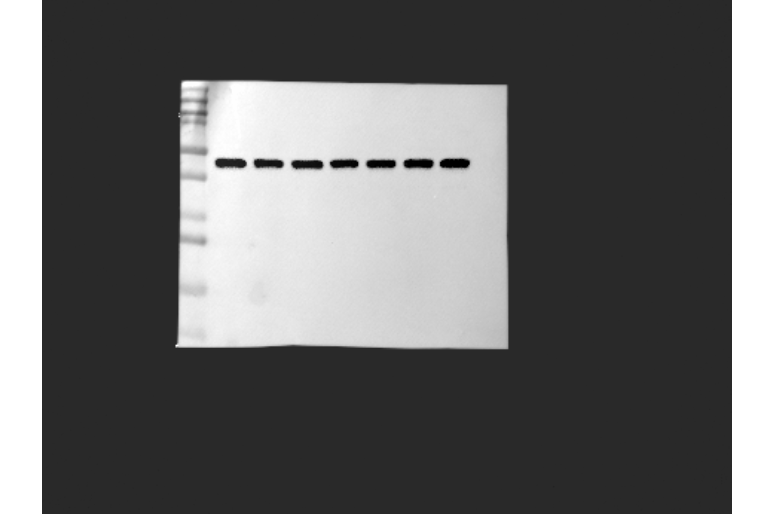

Supplement: Supplementary file 9 [file DataSheet2.zip › actin 2.tif]

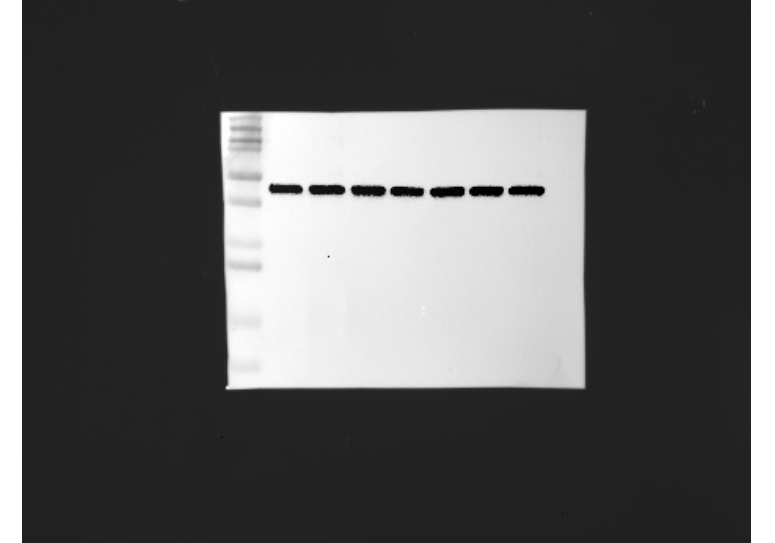

Supplement: Supplementary file 9 [file DataSheet2.zip › actin 3.tif]

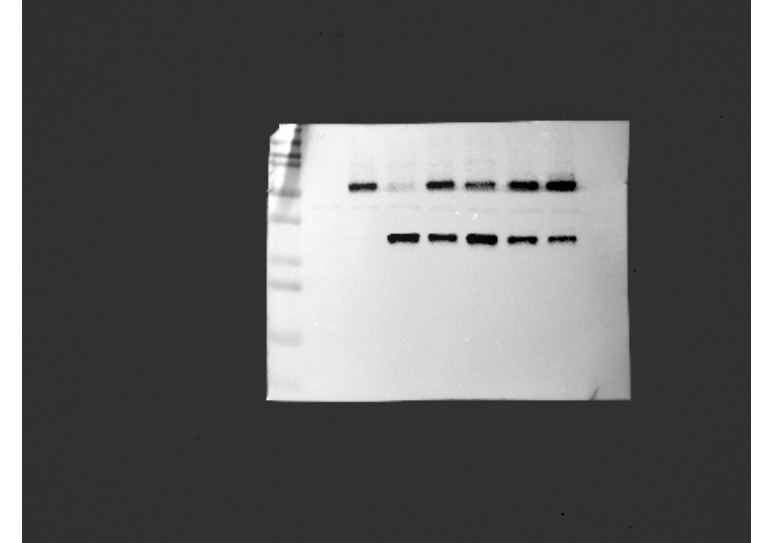

Supplement: Supplementary file 9 [file DataSheet2.zip › p53 1.tif]

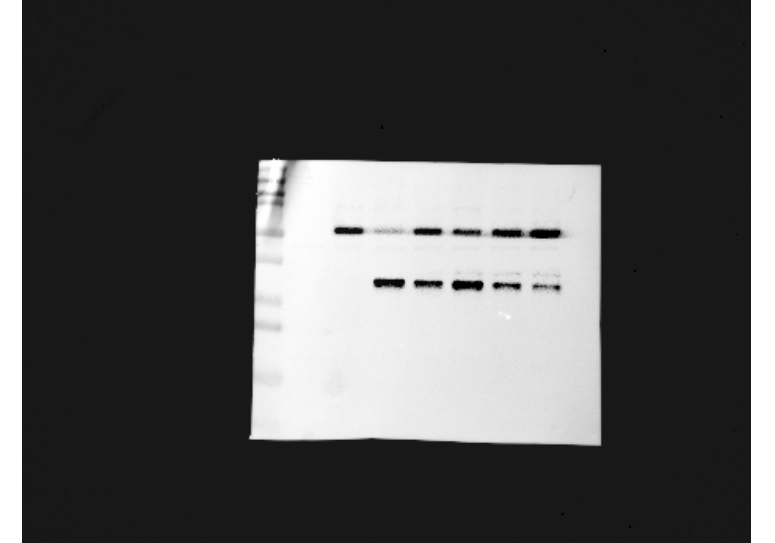

Supplement: Supplementary file 9 [file DataSheet2.zip › p53 2.tif]

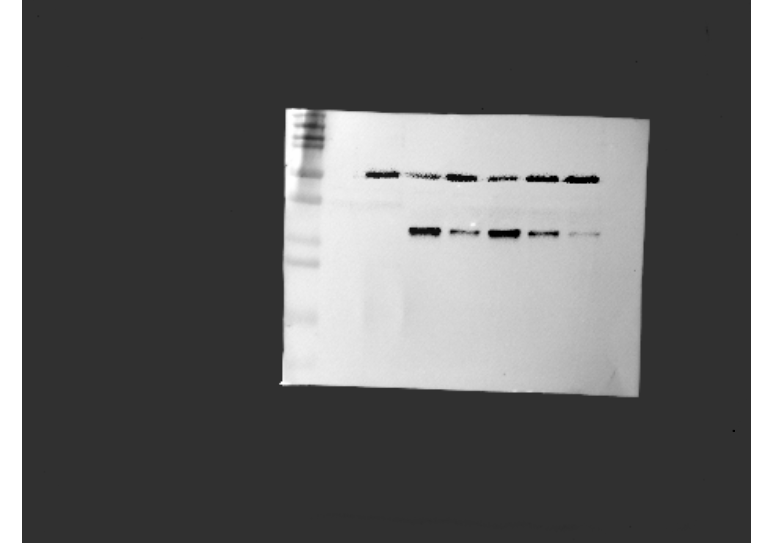

Supplement: Supplementary file 9 [file DataSheet2.zip › p53 3.tif]

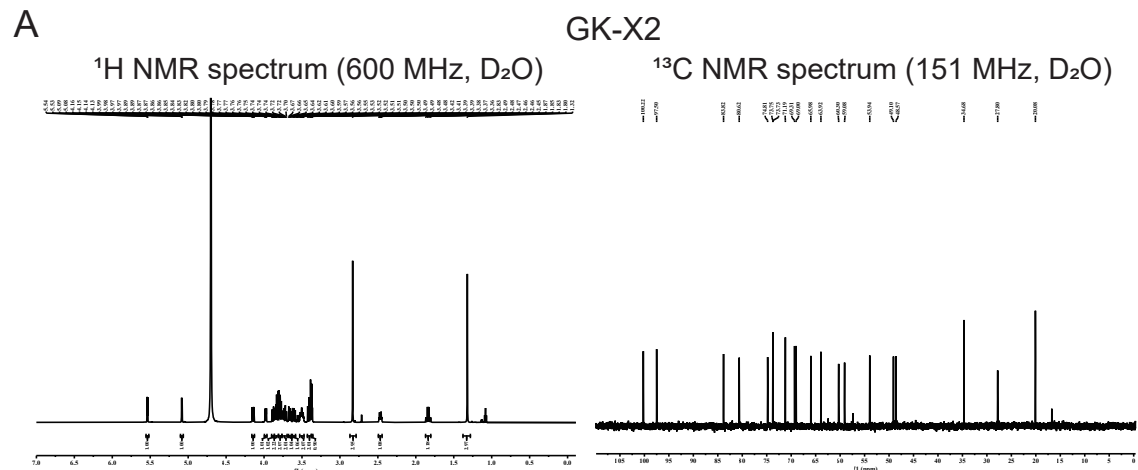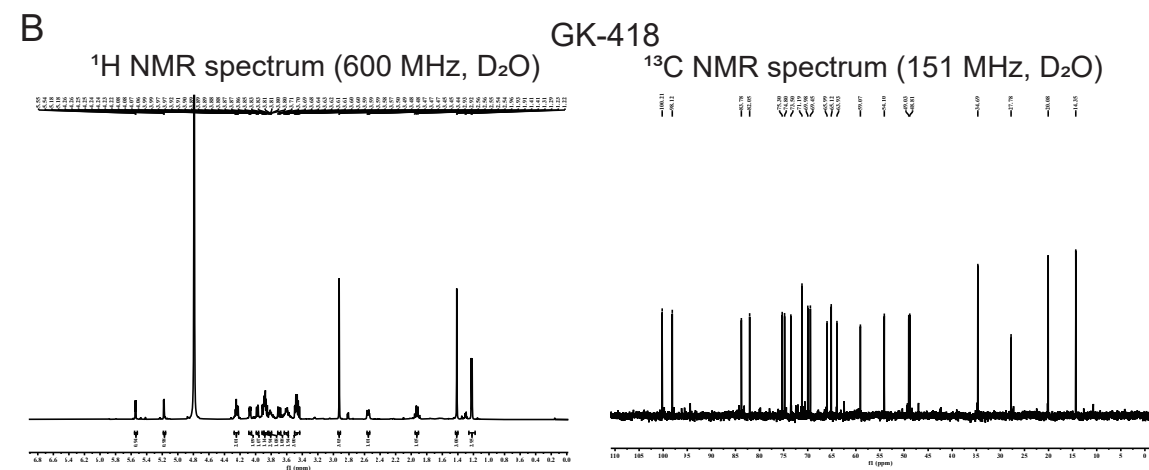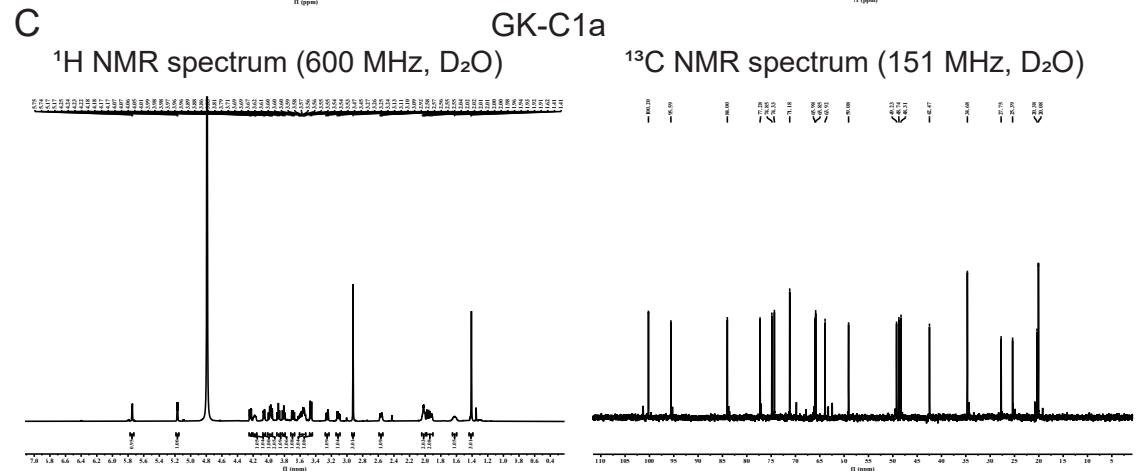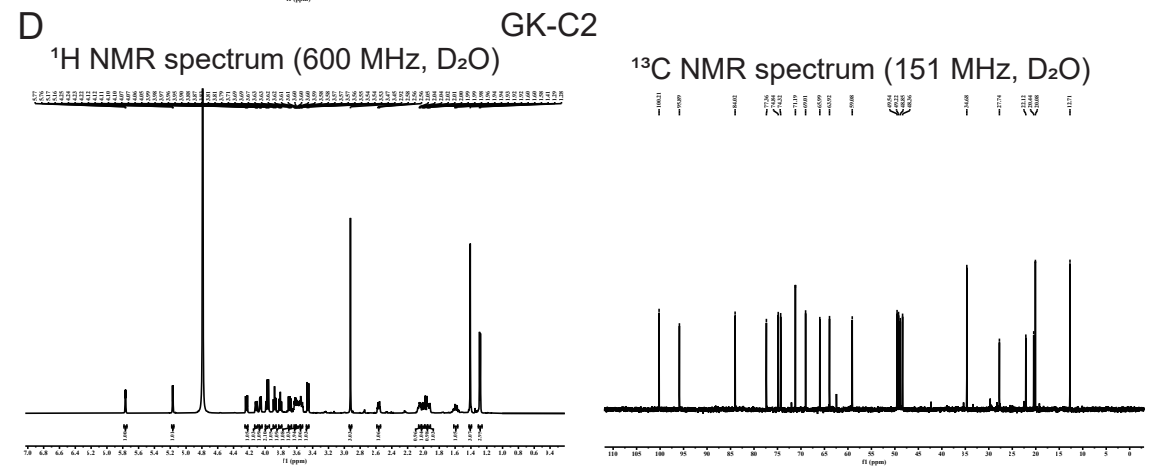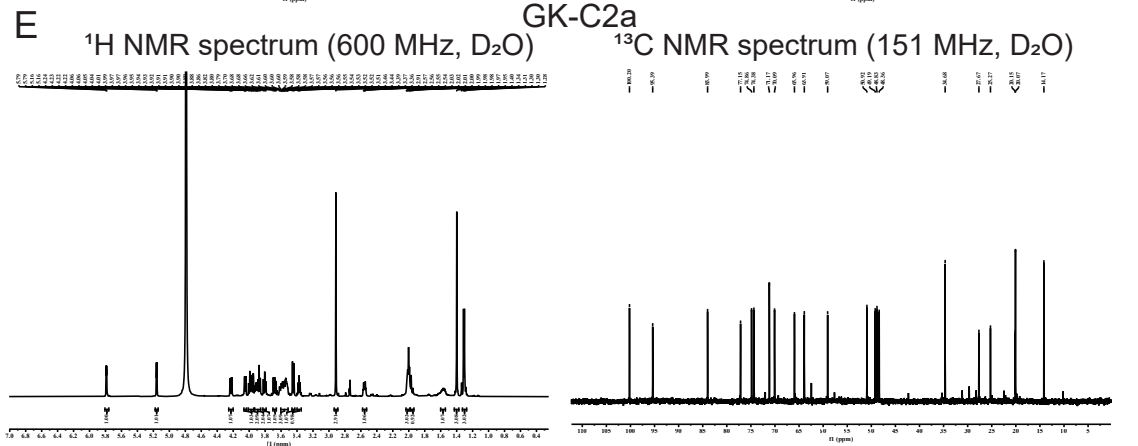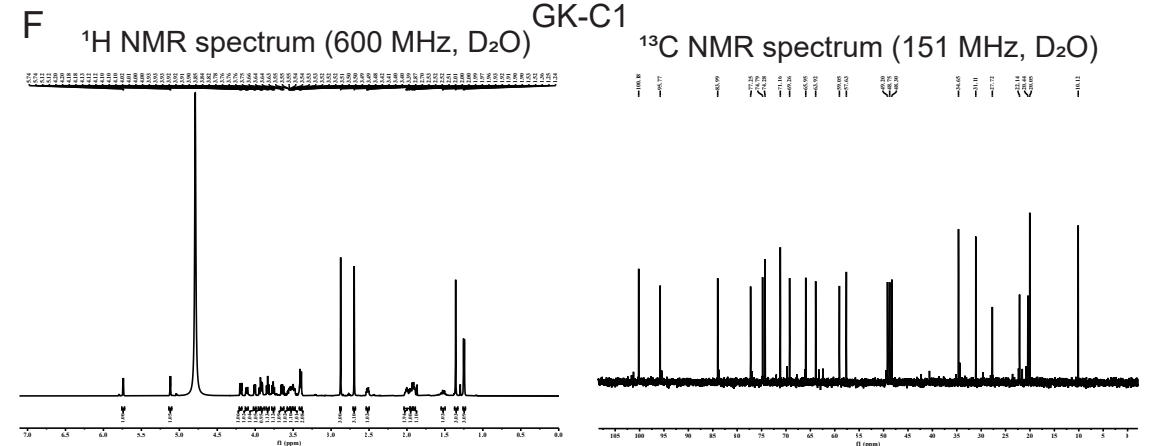

Supplement: Supplementary file 11 [file Image7.pdf]

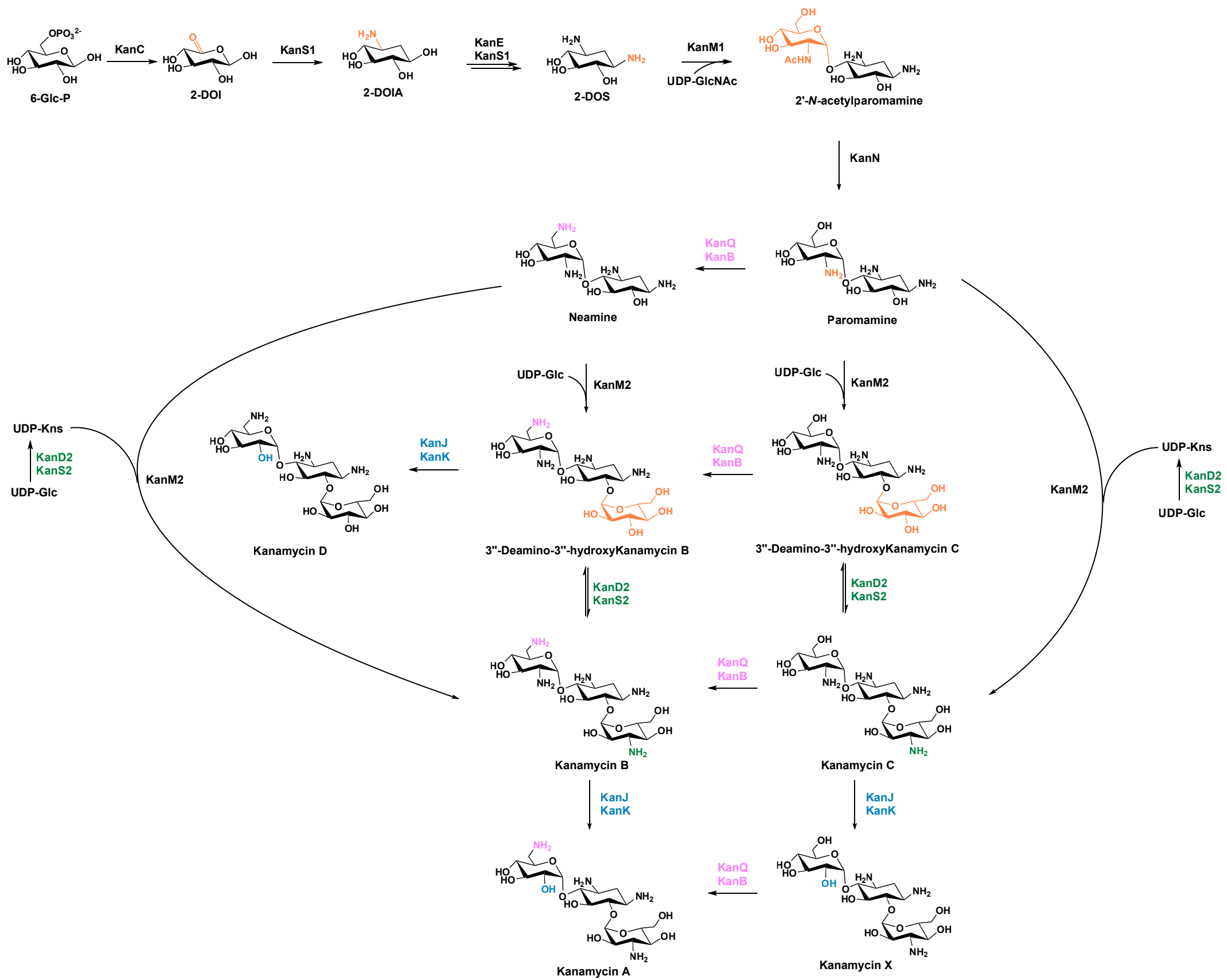

Supplement: Supplementary file 13 [file Image1.pdf]
