# Supplementary material for: Combinatorial biosynthesis of novel gentamicin derivatives with nonsense mutation readthrough activity and low cytotoxicity
Source: Front Pharmacol. 2025 Apr 24;16:1575840. doi: 10.3389/fphar.2025.1575840 (PMC12059486; doi:10.3389/fphar.2025.1575840)
Supplement: Supplementary file 5 [file Image2.pdf]

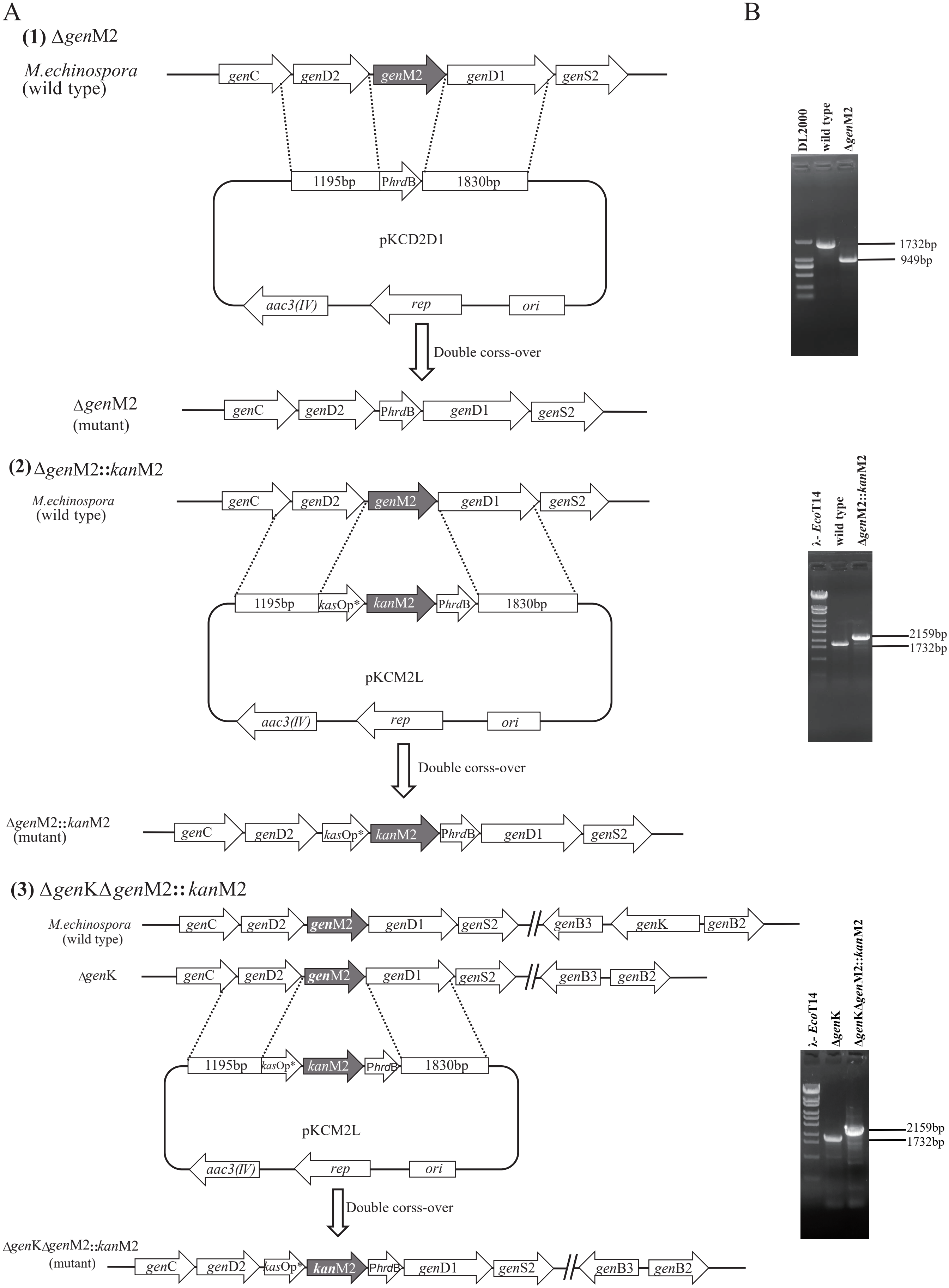
A
(1) ΔgenM2
M.echinospora
(wild type)
genC
genD2
genM2
genD1
genS2
1195bp
PhrdB
1830bp
pKCD2D1
aac3(IV)
rep
ori
Double corss-over
ΔgenM2
(mutant)
genC
genD2
PhrdB
genD1
genS2
(2) ΔgenM2::kanM2
M.echinospora
(wild type)
genC
genD2
genM2
genD1
genS2
1195bp
kasOp*
kanM2
PhrdB
1830bp
pKCM2L
aac3(IV)
rep
ori
Double corss-over
ΔgenM2::kanM2
(mutant)
genC
genD2
kasOp*
kanM2
PhrdB
genD1
genS2
(3) ΔgenKΔgenM2::kanM2
M.echinospora
(wild type)
genC
genD2
genM2
genD1
genS2
genB3
genK
genB2
ΔgenK
genC
genD2
genM2
genD1
genS2
genB3
genB2
1195bp
kasOp*
kanM2
PhrdB
1830bp
pKCM2L
aac3(IV)
rep
ori
Double corss-over
ΔgenKΔgenM2::kanM2
(mutant)
genC
genD2
kasOp*
kanM2
PhrdB
genD1
genS2
genB3
genB2
B
DL2000
wild type
ΔgenM2
1732bp
949bp
λ- EcoT14
wild type
ΔgenM2::kanM2
2159bp
1732bp
λ- EcoT14
ΔgenK
ΔgenKΔgenM2::kanM2
2159bp
1732bp
