# Supplementary material for: Combinatorial biosynthesis of novel gentamicin derivatives with nonsense mutation readthrough activity and low cytotoxicity
Source: Front Pharmacol. 2025 Apr 24;16:1575840. doi: 10.3389/fphar.2025.1575840 (PMC12059486; doi:10.3389/fphar.2025.1575840)
Supplement: Supplementary file 7 [file Table2.docx]

Supplementary Table 2. The bacterial strains and plasmids used in this study.

| Strains/Plasmids | Description | Reference |
| --- | --- | --- |
| Strains |  |  |
| *E. coli* TOP10 | Host for general cloning | Invitrogen |
| *E. coli* ET12567 | Donor strain for conjugation between *E. coli* and *Streptomyces* | (1) |
| *M. echinospora* | Wild-type strain, gentamicin C1a, C2, C2a, C1 producer | ATCC15835 |
| *S. kanamyceticus* | Wild-type strain, kanamycin A, B, C producer | CGMCC4.1441 |
| △*gen*K | *M. echinospora* with *gen*K disrupted | (2) |
| △*gen*M2 | *M. echinospora* with *gen*M2 disrupted | This study |
| △*gen*K△*gen*M2 | *M. echinospora* with *gen*K and *gen*M2 disrupted | This study |
| △*gen*M2::*kan*M2 | Complementation of *kan*M2 in △*gen*M2 | This study |
| △*gen*K△*gen*M2::*kan*M2 | Complementation of *kan*M2 in △*gen*K△*gen*M2 | This study |
| △*gen*M2△*gen*D1::*kan*M2 | GK-Ae accumulating mutant | This study |
| △*gen*K△*gen*M2△*gen*D1::*kan*M2 | GK-A accumulating mutant | This study |
| Plasmids |  |  |
| pPT2925 | pIJ2925 derivative carrying the P*hrd*B promoter and To terminator | This lab |
| pJY813 | *E. coli* - *Streptomyces* shuttle vector carrying *kas*Op* promoter | This lab |
| pKC1139 | *E. coli-streptomyces* shuttle vector | (3) |
| pKCD2D1 | Construct for *gen*M2 disruption | This study |
| pKCM2L | Construct for *kan*M2 complementation | This study |
| pKCM2LS2 | Construct for *kan*M2 complementation and *gen*M2-*gen*D1 disruption | This study |

**References**

1. MacNeil DJ, Gewain KM, Ruby CL, Dezeny G, Gibbons PH, MacNeil T. Analysis of Streptomyces avermitilis genes required for avermectin biosynthesis utilizing a novel integration vector. Gene. 1992;111(1):61-8.

2. Li D, Li H, Ni X, Zhang H, Xia H. Construction of a gentamicin C1a-overproducing strain of Micromonospora purpurea by inactivation of the gacD gene. Microbiol Res. 2013;168(5):263-7.

3. Bierman M, Logan R, O'Brien K, Seno ET, Rao RN, Schoner BE. Plasmid cloning vectors for the conjugal transfer of DNA from Escherichia coli to Streptomyces spp. Gene. 1992;116(1):43-9.
