# Supplementary material for: Combinatorial biosynthesis of novel gentamicin derivatives with nonsense mutation readthrough activity and low cytotoxicity
Source: Front Pharmacol. 2025 Apr 24;16:1575840. doi: 10.3389/fphar.2025.1575840 (PMC12059486; doi:10.3389/fphar.2025.1575840)
Supplement: Supplementary file 8 [file Table3.docx]

Supplementary Table 3. Oligonucleotide primers used in this study.

| Primer | Oligonucleotide sequences (5’ to 3’) | Restriction site |
| --- | --- | --- |
| genM2-L-F | GGGAATTCGCGAGGTCATCAACGAGATC | *Eco*RI |
| genM2-L-R | GCTCTAGACCGGATATCGCGTAGAGGAAGAACGGTGTC | *Xba*I, *Eco*RV |
| genM2-R-F | CATGCCATGGCCGTCACTAAAAAGATCG | *Nco*I |
| genM2-R-R | CCGCTCGAGAAGCTTAGTAGTTCTCCAATGCGAAGG | *Xho*I, *Hin*dIII |
| kanM2-F | GGCATATGACCGAGCCTGCCAAGGGTG | *Nde*I |
| kanM2-R | GCTCTAGAAGATCTTCACAGCCCGATCTCCCGGTAG | *Xba*I |
| KasOp-F | CCGGATATCGGAACGATCGTTGGCTGTGTTC | *Eco*RV |
| KasOp-R | GCAGGTGCATGCCGCCCATATGGCGTATCCCC | NdeI |
| genD1-R-F | CCATGGCGCAGAAACTGGC | *Nco*I |
| genD1-R-R | CTCGAGAAGCTTGCGGTGATGTGGAACAG | *Xho*I, *Hin*dIII |
| genM2-C-F | GATGAATGCCTGACAACAAG |  |
| genM2-C-R | TGGTAGAAGGCGTGGTAG |  |
| genD1-C-R | GTTGTCGTCGGAGATGTC |  |
| WT-F | AGAAACACTTTTCGACATAGTGTGGCTTCCAAGGTGTACGACCC |  |
| WT-R | CACACTATGTCGAAAAGTGTTTCTCATGGTGGCTAGCCTATAGTGAGTC |  |
| 213X-F | AGAAACACTTTTTGACATAGTGTGGCTTCCAAGGTGTACGACCC |  |
| 213X-R | CACACTATGTCAAAAAGTGTTTCTCATGGTGGCTAGCCTATAGTGAGTC |  |
| P53-F | GCGCTTCGAGATGTTCCGAGA |  |
| P53-R | AGTCTGAGTCAGGCCCTTCT |  |
| BAX-F | GCTGTTGGGCTGGATCCAAG |  |
| BAX-R | TCAGCCCATCTTCTTCCAGA |  |
| P21-F | GTGGACCTGTCACTGTCTTGTA |  |
| P21-R | GCGTTTGGAGTGGTAGAAATCTGTC |  |
| Actin-F | CTCCATCCTGGCCTCGCTGT |  |
| Actin-R | GCTGTCACCTTCACCGTTCC |  |
