# Supplementary material for: Combinatorial biosynthesis of novel gentamicin derivatives with nonsense mutation readthrough activity and low cytotoxicity
Source: Front Pharmacol. 2025 Apr 24;16:1575840. doi: 10.3389/fphar.2025.1575840 (PMC12059486; doi:10.3389/fphar.2025.1575840)
Supplement: Supplementary file 10 [file Table4.docx]

Supplementary Table 4. ^1^H NMR (600 MHz, D_2_O) and ^13^C NMR (151 MHz, D_2_O) data

| Products | Site | δ_C_ | δ_H_ |
| --- | --- | --- | --- |
| GK-A | 1 | 50.44, CH | 3.00, ddd (12.4, 9.7, 4.2) |
|  | 2 | 34.62, CH_2_ | 2.02, dt (13.0, 4.3) |
|  |  |  | 1.31, q (12.5) |
|  | 3 | 49.35, CH | 2.93, ddd (12.1, 9.6, 4.2) |
|  | 4 | 86.47, CH | 3.34, m |
|  | 5 | 74.26, CH | 3.67, m |
|  | 6 | 87.18, CH | 3.34, m |
|  | 1' | 100.28, CH | 5.30, d (3.8) |
|  | 2' | 55.26, CH | 2.84, dd (10.4, 3.8） |
|  | 3' | 73.39, CH | 3.60, m |
|  | 4' | 69.89, CH | 3.40, t (8.5) |
|  | 5' | 73.03, CH | 3.86, m |
|  | 6' | 60.72, CH_2_ | 3.88, m |
|  |  |  | 3.74, m |
|  | 1'' | 100.09, CH | 5.09, d (3.8) |
|  | 2'' | 68.54, CH | 3.84, m |
|  | 3'' | 61.36, CH | 3.09, t (10.4) |
|  | 4'' | 65.82, CH | 3.65, m |
|  | 5'' | 72.28, CH | 3.96, dt (10.0, 3.4) |
|  | 6'' | 59.99, CH_2_ | 3.79, m |
|  |  |  | 3.79, m |
|  | C-3''-N-CH_3_ | 31.63, CH_3_ | 2.61, s |
| GK-Ae | 1 | 50.47, CH | 2.97, m |
|  | 2 | 34.87, CH_2_ | 2.00, dt (13.0, 4.2) |
|  |  |  | 1.28, q |
|  | 3 | 49.34, CH | 2.91, m |
|  | 4 | 86.75, CH | 3.34, m |
|  | 5 | 74.19, CH | 3.67, t (9.2) |
|  | 6 | 87.27, CH | 3.30, m |
|  | 1' | 100.32, CH | 5.27, d (3.9) |
|  | 2' | 55.30, CH | 2.81, dd (10.0, 3.4) |
|  | 3' | 73.75, CH | 3.58, m |
|  | 4' | 71.08, CH | 3.34, m |
|  | 5' | 74.77, CH | 3.87, dd (10.2, 2.7) |
|  | 6' | 66.18, CH | 4.17, m |
|  | 1'' | 100.09, CH | 5.07, d (3.9) |
|  | 2'' | 69.01, CH | 3.74, dd (10.8, 3.8） |
|  | 3'' | 61.42, CH | 2.99, d (10.2) |
|  | 4'' | 66.37, CH | 3.61, m |
|  | 5'' | 72.23, CH | 3.96, dt (10.0, 3.4) |
|  | 6'' | 60.05, CH_2_ | 3.78, d (3.4) |
|  |  |  | 3.78, d (3.4) |
|  | C-6'-CH_3_ | 14.95, CH_3_ | 1.21, d (6.6) |
|  | C-3''-N-CH_3_ | 32.15, CH_3_ | 2.55, s |
| GK-X2 | 1 | 49.10, CH | 3.51, m |
|  | 2 | 27.80, CH_2_ | 2.47, dt (12.6, 4.3) |
|  |  |  | 1.84, q (12.7) |
|  | 3 | 48.57, CH | 3.40, m |
|  | 4 | 80.62, CH | 3.74, m |
|  | 5 | 73.75, CH | 3.79, m |
|  | 6 | 83.82, CH | 3.74, m |
|  | 1' | 97.50, CH | 5.54, d (4.0) |
|  | 2' | 53.94, CH | 3.37, d (3.6) |
|  | 3' | 69.00, CH | 3.84, m |
|  | 4' | 69.31, CH | 3.40, m |
|  | 5' | 73.73, CH | 3.79, m |
|  | 6' | 60.30, CH_2_ | 3.84, m |
|  |  |  | 3.66, m |
|  | 1'' | 100.22, CH | 5.08, d (3.9) |
|  | 2'' | 65.98, CH | 4.14, dd (11.0, 3.9） |
|  | 3'' | 63.92, CH | 3.51, m |
|  | 4'' | 71.19, C | / |
|  | 5'' | 74.81, CH | 3.98, dd (8.7, 2.7) |
|  | 6'' | 59.08, CH_2_ | 3.88, dd (11.8, 2.7) |
|  |  |  | 3.61, dd (11.8, 8.7) |
|  | C-3''-N-CH_3_ | 34.68, CH_3_ | 2.83, s |
|  | C-4''-CH_3_ | 20.08, CH_3_ | 1.32, s |
| GK-418 | 1 | 49.03, CH | 3.60, m |
|  | 2 | 27.78, CH_2_ | 2.55, m |
|  |  |  | 1.92, d (12.7) |
|  | 3 | 48.81, CH | 3.60, m |
|  | 4 | 82.05, CH | 3.91, m |
|  | 5 | 73.5, CH | 3.87, m |
|  | 6 | 83.78, CH | 3.81, m |
|  | 1' | 98.12, CH | 5.54, d (4.1) |
|  | 2' | 54.1, CH | 3.47, m |
|  | 3' | 69.45, CH | 3.87, m |
|  | 4' | 69.98, CH | 3.47, m |
|  | 5' | 75.3, CH | 3.87, m |
|  | 6' | 65.12, CH | 4.25, m |
|  | 1'' | 100.21, CH | 5.18, d (3.9) |
|  | 2'' | 65.99, CH | 4.25, m |
|  | 3'' | 63.93, CH | 3.47, m |
|  | 4'' | 71.19, C | / |
|  | 5'' | 74.8, CH | 4.07, dd (8.7, 2.7) |
|  | 6'' | 59.07, CH_2_ | 3.98, dd (11.8, 2.7) |
|  |  |  | 3.70, dd (11.9, 8.7) |
|  | C-6'-CH_3_ | 14.35, CH_3_ | 1.22, d (6.6) |
|  | C-3''-N-CH_3_ | 34.69, CH_3_ | 2.92, s |
|  | C-4''-CH_3_ | 20.08, CH_3_ | 1.41, s |
| GK-C1a | 1 | 49.23, CH | 3.57, m |
|  | 2 | 27.75, CH_2_ | 2.57, dt (12.7, 4.3) |
|  |  |  | 1.94, m |
|  | 3 | 48.31, CH | 3.57, m |
|  | 4 | 77.28, CH | 3.98, m |
|  | 5 | 74.33, CH | 3.88, t (9.0) |
|  | 6 | 84.00, CH | 3.81, t (9.5) |
|  | 1' | 95.59, CH | 5.75, d (3.6) |
|  | 2' | 48.74, CH | 3.57, m |
|  | 3' | 20.38, CH_2_ | 2.02, m |
|  |  |  | 2.02, m |
|  | 4' | 25.39, CH_2_ | 1.94, m |
|  |  |  | 1.62, m |
|  | 5' | 65.98, CH | 4.18, m |
|  | 6' | 42.47, CH_2_ | 3.25, dd (13.6, 3.5) |
|  |  |  | 3.11, dd (3.5, 7.0 |
|  | 1'' | 100.20, CH | 5.17, d (3.9) |
|  | 2'' | 65.85, CH | 4.24, dd (11.0, 3.9) |
|  | 3'' | 63.92, CH | 3.46, d (11.0) |
|  | 4'' | 71.18, C | / |
|  | 5'' | 74.85, CH | 4.06, dd (8.6, 2.6) |
|  | 6'' | 59.08, CH_2_ | 3.98, m |
|  |  |  | 3.69, dd (11.8, 8.7) |
|  | C-3''-N-CH_3_ | 34.68, CH_3_ | 2.92, s |
|  | C-4''-CH_3_ | 20.08, CH_3_ | 1.41, s |
| GK-C2 | 1 | 49.22, CH | 3.62, m |
|  | 2 | 27.74, CH_2_ | 2.57, dt (12.6, 4.3) |
|  |  |  | 1.97, d (12.6) |
|  | 3 | 48.85, CH | 3.59, m |
|  | 4 | 77.36, CH | 3.97, m |
|  | 5 | 74.32, CH | 3.88, t (9.0) |
|  | 6 | 84.02, CH | 3.81, t (8.8) |
|  | 1' | 95.89, CH | 5.77, d (3.6) |
|  | 2' | 48.36, CH | 3.59, m |
|  | 3' | 20.44, CH_2_ | 2.04, dt (8.3, 4.1) |
|  |  |  | 2.00, m |
|  | 4' | 22.12, CH_2_ | 1.93, m |
|  |  |  | 1.59, m |
|  | 5' | 69.01, CH | 4.11, dt (12.3, 3.0) |
|  | 6' | 49.54, CH | 3.54, m |
|  | 1'' | 100.21, CH | 5.17, d (4.0) |
|  | 2'' | 65.99, CH | 4.24, dd (11.0, 3.9) |
|  | 3'' | 63.92, CH | 3.46, d (11.0) |
|  | 4'' | 71.19, C | / |
|  | 5'' | 74.84, CH | 4.06, dd (8.7, 2.6) |
|  | 6'' | 59.08, CH | 3.97, m |
|  |  |  | 3.69, dd (11.8, 8.7) |
|  | C-6'-CH_3_ | 12.71, CH_3_ | 1.29, d (7.0) |
|  | C-3''-N-CH_3_ | 34.68, CH_3_ | 2.92, s |
|  | C-4''-CH_3_ | 20.08, CH_3_ | 1.41, s |
| GK-C2a | 1 | 49.19, CH | 3.65, m |
|  | 2 | 27.67, CH_2_ | 2.56, dt (12.7, 4.3) |
|  |  |  | 1.96, d (12.7) |
|  | 3 | 48.83, CH | 3.56, m |
|  | 4 | 77.15, CH | 4.05, dd (8.7, 2.6) |
|  | 5 | 74.38, CH | 3.89, m |
|  | 6 | 83.99, CH | 3.89, m |
|  | 1' | 95.39, CH | 5.79, d (3.6) |
|  | 2' | 48.36, CH | 3.56, m |
|  | 3' | 20.15, CH_2_ | 2.01, m |
|  |  |  | 2.01, m |
|  | 4' | 25.27, CH_2_ | 2.01, m |
|  |  |  | 1.54, m |
|  | 5' | 70.09, CH | 3.81, t (9.5) |
|  | 6' | 50.92, CH | 3.37, m |
|  | 1'' | 100.2, CH | 5.16, d (4.0) |
|  | 2'' | 65.96, CH | 4.23, dd (11.0, 3.9) |
|  | 3'' | 63.91, CH | 3.45, d (11.0) |
|  | 4'' | 71.17, C | / |
|  | 5'' | 74.86, CH | 3.97, m |
|  | 6'' | 59.07, CH_2_ | 3.97, m |
|  |  |  | 3.68, dd (11.9, 8.7) |
|  | C-6'-CH_3_ | 14.17, CH_3_ | 1.31, d (6.8) |
|  | C-3''-N-CH_3_ | 34.68, CH_3_ | 2.91, s |
|  | C-4''-CH_3_ | 20.07, CH_3_ | 1.40, s |
| GK-C1 | 1 | 49.2, CH | 3.55, m |
|  | 2 | 27.72, CH_2_ | 2.52, dt (12.7, 4.3) |
|  |  |  | 1.93, m |
|  | 3 | 48.75, CH | 3.49, m |
|  | 4 | 77.25, CH | 3.93, m |
|  | 5 | 74.28, CH | 3.84, t (9.0) |
|  | 6 | 83.99, CH | 3.76, t (8.8) |
|  | 1' | 95.77, CH | 5.74, d (3.5) |
|  | 2' | 48.3, CH | 3.52, m |
|  | 3' | 20.44, CH_2_ | 2.00, m |
|  |  |  | 2.00, m |
|  | 4' | 22.14, CH_2_ | 1.91, m |
|  |  |  | 1.53, m |
|  | 5' | 69.26, CH | 4.11, dt (12.3, 2.8) |
|  | 6' | 57.63, CH | 3.4, m |
|  | 1'' | 100.18, CH | 5.12, d (3.9) |
|  | 2'' | 65.95, CH | 4.19, dd (11.0, 3.9) |
|  | 3'' | 63.92, CH | 3.4, m |
|  | 4'' | 71.16, C | / |
|  | 5'' | 74.79, CH | 4.01, dd (8.7, 2.6) |
|  | 6'' | 59.05, CH_2_ | 3.91, m |
|  |  |  | 3.64, dd (11.8, 8.7) |
|  | C-6'-CH_3_ | 34.65, CH_3_ | 2.87, s |
|  | C-6'-N-CH_3_ | 20.05, CH_3_ | 1.36, s |
|  | C-3''-N-CH_3_ | 31.11, CH_3_ | 2.7, s |
|  | C-4''-CH_3_ | 10.12, CH_3_ | 1.41, s |
