# Supplementary material for: Combinatorial biosynthesis of novel gentamicin derivatives with nonsense mutation readthrough activity and low cytotoxicity
Source: Front Pharmacol. 2025 Apr 24;16:1575840. doi: 10.3389/fphar.2025.1575840 (PMC12059486; doi:10.3389/fphar.2025.1575840)
Supplement: Supplementary file 12 [file DataSheet1.docx]

**Captions for supplementary figures**

**Supplementary Figure 1. Biosynthetic pathway of kanamycins.** The structural changes are marked in orange. The functional groups and the corresponding enzymes are indicated by the same color, respectively. Abbreviations: 6-Glc-P, glucose 6-phosphate; 2-DOI, 2-deoxy-scyllo-inosose; 2-DOIA, 2-deoxy-scyllo-inosamine; 2-DOS, 2-deoxystreptamine; UDP-GlcNAc, uridine 5′-diphospho-D-2-N-acetylglucosamine; UDP-Glc, UDP-glucose; UDP-kns, UDP-kanosamine.

**Supplementary Figure 2. Construction of *gen*M2 deletion and *kan*M2 expression mutants.** (A) Schematic diagram of the in-frame deletion. (B) PCR confirmation of in-frame gene deletion mutant with primers genM2-C-F/genM2-C-R in Supplementary Table 2.

**Supplementary Figure 3. Construction of *kan*M2 expression mutants.** (A) Schematic diagram of the in-frame deletion. (B) PCR confirmation of in-frame gene deletion mutant with primers genM2-C-F/genD1-C-R in Supplementary Table 2.

**Supplementary Figure 4. LC-ESI-HRMS and MS/MS analysis of products in Δ*gen*M2::*kan*M2.** (A-I) LC-ESI-HRMS and MS/MS analysis of products in Δ*gen*M2::*kan*M2, including GK-A (A), GK-X2 (B), GK-Ae (C), GK-418 (D), GK-C1a (E), GK-C2 (F), GK-C2b (G), GK-C2a (H) and GK-C1 (I).

**Supplementary Figure 5. Spectrum analysis on GK-A.** (A) ^1^H NMR spectrum (600 MHz, D_2_O) of GK-A. (B) ^1^³C NMR spectrum (151 MHz, D_2_O) of GK-A. (C) HSQC spectrum (600 MHz, D_2_O) of GK-A. (D) ^1^H–^1^H COSY spectrum (600 MHz, D_2_O) of GK-A. (E) HMBC spectrum (600 MHz, D_2_O) of GK-A. (F) NOESY spectrum (600 MHz, D_2_O) of GK-A.

**Supplementary Figure 6. Spectrum analysis on GK-Ae.** (A) ^1^H NMR spectrum (600 MHz, D_2_O) of GK-Ae. (B) ^1^³C NMR spectrum (151 MHz, D_2_O) of GK-Ae. (C) HSQC spectrum (600 MHz, D_2_O) of GK-Ae. (D) ^1^H–^1^H COSY spectrum (600 MHz, D_2_O) of GK-Ae. (E) HMBC spectrum (600 MHz, D_2_O) of GK-Ae. (F) NOESY spectrum (600 MHz, D_2_O) of GK-Ae.

**Supplementary Figure 7. ^1^H NMR spectrum and ^13^C NMR spectrum of products in Δ*gen*M2::*kan*M2.** (A) ^1^H NMR spectrum and ^13^C NMR spectrum of GK-X2. (B) ^1^H NMR spectrum and ^13^C NMR spectrum of GK-418. (C) ^1^H NMR spectrum and ^13^C NMR spectrum of GK-C1a. (D) ^1^H NMR spectrum and ^13^C NMR spectrum of GK-C2. (E) ^1^H NMR spectrum and ^13^C NMR spectrum of GK-C2a. (F) ^1^H NMR spectrum and ^13^C NMR spectrum of GK-C1.
